# Ultrasound-Guided Versus Palpation-Guided Techniques to Achieve Vascular Access in Children Undergoing Cardiac Surgery: A Systematic Review and Meta-analysis of Randomized Controlled Trials

**DOI:** 10.1007/s00246-024-03581-y

**Published:** 2024-07-27

**Authors:** Ahmed A. Ibrahim, Abdallah R. Allam, Ahmed Mazen Amin, Mohamed Saad Rakab, Abdulhameed Alhadeethi, Ahmed W. Hageen, Abdelrahman Mahmoud, Mohamed Abuelazm, Basel Abdelazeem

**Affiliations:** 1 Faculty of Medicine, Menoufia University, Menoufia, Egypt; 2 Faculty of Medicine, Mansoura University, Mansoura, Egypt; 3 Department of General Medicine, Al-Salam Teaching Hospital, Ninevah, Iraq; 4 Faculty of Medicine, Tanta University, Tanta, Egypt; 5 Faculty of Medicine, Minia University, Minia, Egypt; 6 Department of Cardiology, West Virginia University, Morgantown, WV USA

**Keywords:** Cardiac surgery, Pediatrics, Ultrasound, Cannula, Catheter, Review

## Abstract

Pediatric heart surgery is a vital therapeutic option for congenital heart disease, which is one of the most prevalent causes of death in children. Arterial cannulation (AC) and central venous catheter (CVC) are required in pediatric cardiac surgery for continuous monitoring of the central venous pressure (CVP), replacement of fluid or blood products, close hemodynamic monitoring, and frequent sampling for arterial blood gases (ABG). A systematic review and meta-analysis synthesizing evidence from randomized controlled trials (RCTs) retrieved from PubMed, Embase Cochrane, Scopus, and WOS until February 2024. Risk ratio (RR) was used to report dichotomous outcomes, and mean difference (MD) was used to report continuous outcomes, both with a 95% confidence interval (CI) using the random-effects model. Thirteen RCTs with 1060 children were included. Regarding arterial cannulation, the ultrasound-guided technique (US) was associated with a statistically significant increase in successful cannulation [RR: 1.31 with 95% CI (1.10, 1.56), P < 0.0001], and first-attempt success [RR: 1.88 with 95% CI (1.35, 2.63), P < 0.0001]. However, US was not associated with any statistically significant difference in venous cannulation in both outcomes with [RR: 1.13 with 95% CI (0.98, 1.30), P = 0.10], [RR: 1.53 with 95% CI (0.86, 2.71), P = 0.15] respectively. Moreover, US was associated with a statistically significant decrease in the number of attempts either in arterial cannulation with [MD: − 0.73 with 95% CI (− 1.00, − 0.46), P < 0.0001] or in venous cannulation with [MD: − 1.34 with 95% CI (− 2.55, − 0.12), P = 0.03], and the time of attempted cannulation also either in arterial cannulation with [MD: − 2.27 with 95% CI (− 3.38, − 1.16), P < 0.0001] or in venous cannulation with [MD: − 4.13 with 95% CI (− 7.06, − 1.19), P < 0.0001]. US guidance improves successful cannulation rates and first-attempt success in arterial access and reduces the number of attempts and procedural time for arterial and venous access. It was also associated with a lower incidence of complications and procedure failure, particularly in arterial access. However, it was associated with a higher incidence of venous puncture.

**Supplementary Information:**

The online version contains supplementary material available at 10.1007/s00246-024-03581-y.

## Introduction

Pediatric heart surgery is a vital therapeutic option for congenital heart disease, which is one of the most prevalent causes of death in children [1]. In pediatric cardiac surgery, arterial cannulation (AC) is required for close hemodynamic monitoring and frequent sampling for arterial blood gases (ABG). In addition, a central venous catheter (CVC) is needed for continuous monitoring of the central venous pressure (CVP) and replacement of fluid or blood products [2, 3].

The first choice for AC is the radial artery due to its easy access and fewer complications in children [4, 5]. Nevertheless, the use of radial artery cannulation has been linked to vasospasm. Therefore, a parallel trend has been observed in pediatric patients, where femoral artery catheterization has been associated with enhanced safety standards and accessibility [6–8]. Additionally, previous studies on pediatrics have predominantly focused on catheter line insertions through the internal jugular vein, which was associated with a high success rate and lower complications due to its wide diameter [9].

Traditionally, catheterization is performed using external landmarks and palpation techniques. However, using ultrasound (US) guidance during the femoral artery catheterization proved safer with strong supporting evidence [10]. Additionally, it is the standard of care for central venous catheterization [11–13]. Despite evidence-based recommendations, the adoption of US guidance remains limited, and its endorsement of palpation techniques, particularly in pediatric cases, needs more unequivocal supporting evidence [11, 14, 15]. Moreover, previous research in pediatrics has predominantly focused on catheter line insertions through the internal jugular vein, rendering comparative evidence for femoral artery catheterization techniques [11–16].

Recent investigations have aimed to fill this gap by comparing the success rates and complications associated with US-guided versus palpation-guided techniques in arterial and central venous catheterization in pediatric patients undergoing cardiac surgery. Hence, we conducted this systematic review and meta-analysis to synthesize the available evidence on the safety and efficacy of US-guided versus palpation-guided AC or CVC insertion in pediatric patients undergoing cardiac surgery.

## Methodology

### Protocol Registration

When reporting this systematic review and meta-analysis, we followed the preferred reporting items of systematic reviews and meta-analysis (PRISMA) statement guidelines [17]. We followed the Cochrane Handbook of Systematic Reviews of Interventions [18]. The protocol for this meta-analysis has been registered and published in PROSPERO with the following ID: CRD42024528227. The PRISMA checklist is demonstrated in Table S3.

### Data Sources and Search Strategy

We have established a comprehensive search in various databases such as PubMed, EMBASE, Cochrane (CENTRAL), Scopus, and Web of Science Core Collection, that was systematically approached until the 21st of February 2024, using relevant search terms and keywords, as demonstrated in Table S1.

### Eligibility Criteria

We included randomized controlled trials (RCTs) reported in English that fulfilled the following PICO criteria:

1. *Population*: children undergoing catheterization (cannulation) for cardiac surgery.
2. *Intervention*: US-guided technique in vascular access to the targeted vessel.
3. *Comparison*: palpation (landmark) guided technique.
4. *Outcomes*: our primary outcome was the success rate of cannulation. Secondary outcomes included duration of attempt, number of attempts and used cannula, any complications, artery or vein puncture defined as “An unintentional puncture or perforation of the wall of a blood vessel (artery or vein) during an attempt to cannulate (insert a needle or catheter into) the vessel.”, surgical cutdown, puncture failure, failure to pass the wire, and safety outcomes (mortality and occurrence of any adverse events).

We excluded the following types of articles: (I) studies lacking a comparison group, (II) those containing unreliable, non-extractable, duplicated, or overlapped data sets, (III) articles with unavailable full texts, (IV) conference posters/abstracts, case reports/series, review articles, and protocols of clinical trials with unpublished results.

### Study Selection

This review was achieved using Covidence online software. The obtained studies were independently screened by (A.M., A.A., A.W.H., and M.S.R.) in two phases. The first phase was title/abstract screening for potential clinical studies on Covidence. In the second phase, we retrieved the full-text articles of the selected abstract for further eligibility screening using separate Google sheets. Any conflicts have been resolved by consensus and discussion.

### Data Extraction

Data were extracted by at least two authors of (A.M., A.A., A.W.H., and M.S.R.), using separate Google sheets under three main domains: firstly, the summary characteristics (name of the first author, year of publication, study design, number of centers, country, total participants, venous or arterial canulation, name of the canulated vessel, interventional details, anesthesia used, main inclusion criteria, and primary outcome). The baseline information of the targeted population (sample size, age, weight, height, gender, heart rate, SBP, DBP, and vessel diameter). Eventually, outcome data, as previously illustrated, were included in the third section.

### Risk of Bias and Certainty of Evidence

The quality assessment of studies was independently conducted using the Cochrane RoB2 tool [19] by (A.M., A.A., A.W.H., and M.S.R.). Moreover, they evaluated five domains, including deviation from the intended intervention, the risk of bias linked to the randomization process, outcomes measuring, missing outcome information, and choosing the reported outcomes and results. To evaluate the certainty of the evidence, the Grading of Recommendations Assessment, Development, and Evaluation (GRADE) was utilized [20, 21] by (M.A., B.A.). Any conflicts have been resolved by consensus and discussion.

### Statistical Analysis

For the statistical analysis, R version 4.3 was utilized using meta, metafor, and dmetar packages. Using the random-effects model, we pooled the results of dichotomous outcomes using the risk ratio (RR) and the continuous outcomes using the mean difference (MD), both with a 95% confidence interval (CI). We employed the Chi-square and I-square tests to evaluate heterogeneity; the Chi-square test determines whether heterogeneity exists, while the I-square test evaluates its degree. We considered an alpha level below 0.1 for the Chi-square test to denote significant heterogeneity.

We used both influence analysis and the brute force approach to identify the outlier for the sensitivity analysis. Additionally, our study's heterogeneity patterns were assessed using the Baujat plot. The Baujat plot's (y-axis) displays each effect size’s influence on the pooled result, while the x-axis displays each effect size’s total heterogeneity contribution. Studies or effect sizes that have high values on both the x and y axes could be regarded as influential cases; studies or effect sizes that have a high contribution to heterogeneity (x-axis) but little effect on the overall results could be regarded as outliers and could be eliminated to reduce the amount of heterogeneity between studies.

## Results

### Search Results and Study Selection

**Fig. 1:**
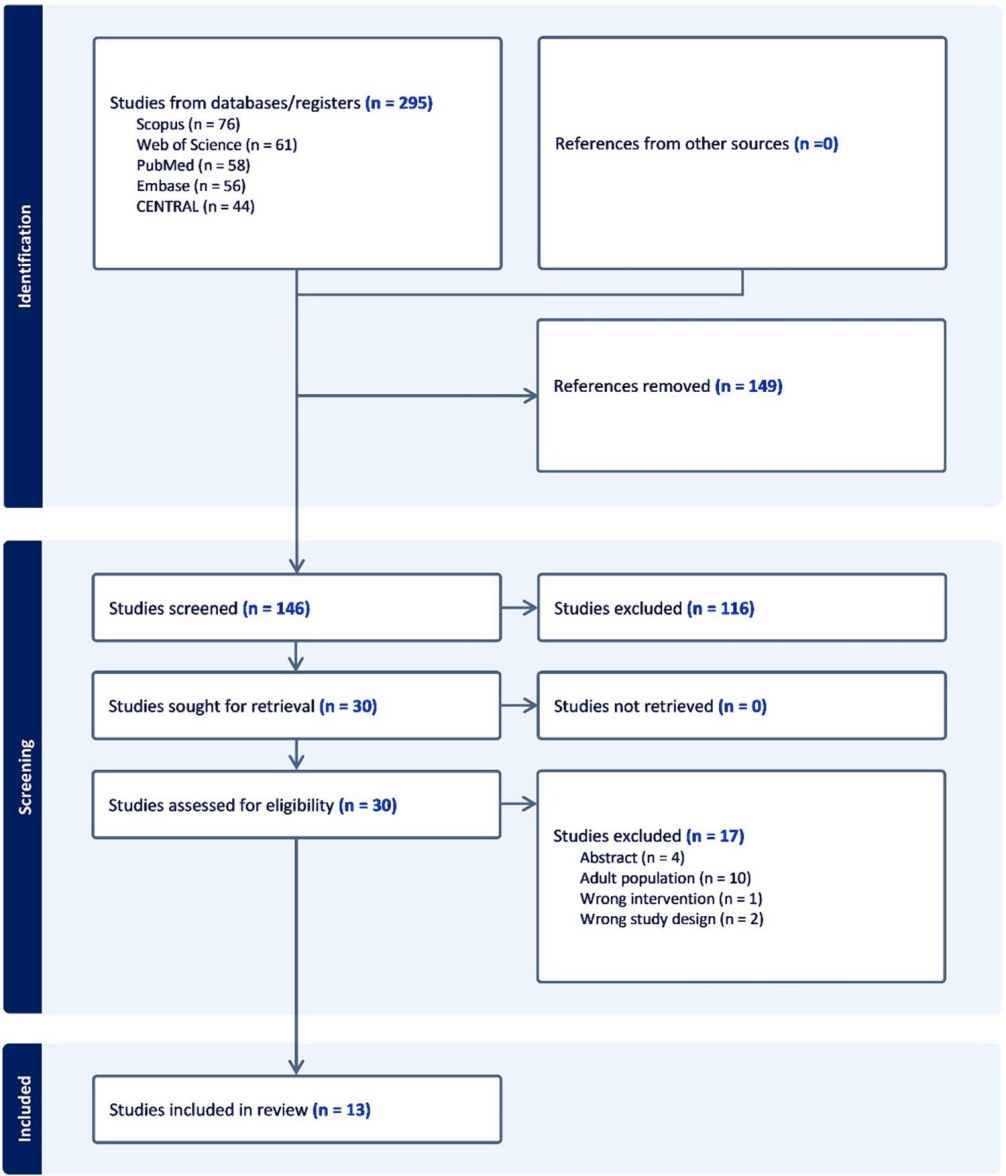
PRISMA flow chart of the screening process

Figure 1 illustrates the process of screening literature. Initially, 295 relevant studies were found by searching databases. After duplicate entries were removed, titles and abstracts were screened to exclude 116 irrelevant articles, and the full texts of the remaining 30 articles were viewed. After the full-text screening, 13 RCTs were included [15, 22–33].

### Characteristics of the Included Studies

A total of 13 RCTs involving 1060 patients fulfilled the inclusion criteria, of which 537 were allocated to the US group and 553 patients to the Palpation Group. These studies varied in the sample size from 40 to 201 and included children who were cannulated with either arterial or venous approach. Tables 1, 2 summarise the main features of the included literature and the baseline characteristics of the included population.

**Table 1:** Summary of the included RCTs

Study	Study design	Country	Total participants	Name of the vessel cannulated	Intervention details		Main inclusion criteria
					U/S group	Palpation group	
Aktiz-Bıçak et al. 2023 (femoral)	Open label- RCT	Turkey	46	Femoral artery	A 5–12 MHz linear probe (Esaote, MyLab Six, Netherlands) was used to perform a femoral artery procedure. The probe, covered with a sterile sheath, located the artery from the inguinal ligament. Confirmation was done with color Doppler and pressure wave functions. The 20-gauge needle puncture was guided by ultrasound, followed by catheter insertion using the Seldinger technique, completing the procedure successfully	A 20-gauge needle was used for arterial puncture, guided by palpating the femoral artery pulse. After confirming blood flow, a guidewire facilitated catheter insertion via the Seldinger technique. The guidewire was removed, and the catheter was secured to the skin	Children in the American Society of Anesthesiologists Physical Status Classification III-IV who underwent surgery for congenital heart surgery between January 2020 and January 2021
Aktiz-Bıçak et al. 2023 (IJV)	Open label- RCT	Turkey	52	IJV	Catheterization was done by a linear probe wrapped in a sterile sheath placed between the cricoid cartilage and clavicle. Optimal positioning for the internal jugular vein (IJV) was achieved through proximal and distal movements. After confirming venous blood aspiration, the Seldinger technique, guided by ultrasound, facilitated guide wire insertion, followed by dilatation and central catheter placement, which secured the catheter in the vein	The entry site for catheterization was determined at the triangle's apex, formed by the sternocleidomastoid muscle heads and clavicle. The needle was directed towards the ipsilateral nipple, and after confirming venous blood aspiration, the Seldinger technique guided guide wire insertion. Dilatation followed, allowing central catheter placement, which was fixed with a 2/0 silk suture	Children in the American Society of Anesthesiologists Physical Status Classification III-IV who underwent surgery for a congenital heart surgery between 1 January 2020 and 1 January 2021
Alderson et al. 1993	Open label- RCT	N/A	40	IJV	The ultrasound scanner was used to visualize the internal jugular vein so that its projection could be marked onto the overlying skin with a pen; this line then served as a guide for cannulation. A 16-gauge polyurethane double-lumen catheter was positioned using a Seldinger wire passed through a 21-gauge needle	The two heads of the sternomastoid were palpated, the position of the carotid artery verified, and the cannula inserted at the junction of the sternomastoid heads angled toward the ipsilateral nipple. A 16-gauge polyurethane double-lumen catheter was positioned using a Seldinger wire passed through a 21-gauge needle	Patients younger than two years who would undergo percutaneous insertion of an internal Jugular cannula during cardiac surgery. None had undergone prior cardiac surgery or jugular cannulation
Aouad et al. 2010	Open-label- RCT	Lebanon	48	Femoral vein	The inguinal area was scanned to identify the femoral artery and vein. Using an out-of-plane technique, the vein was centered on the screen. With the right hand below the US probe at its center, a 20-gauge cannula was introduced while watching for tissue movement on the US screen. The cannula was redirected, or the maneuver was repeated until adequate venous flow was obtained, allowing easy guidewire insertion	A blinded, external landmark-guided technique was used for femoral line insertion. The femoral artery was localized by palpating the pulse in the femoral triangle, and a 20-gauge catheter was inserted medial to the artery. Repeated attempts were made until sufficient venous flow was achieved, enabling the guidewire insertion	Children aged 0 to 12 years, ASA physical status III or IV, with congenital heart disease undergoing cardiac surgery
Grebenik et al. 2004	Open label- RCT	UK	134	IJV	Baird Site-Rite, a three-ultrasound probe with an attached needle guide, was used (within a sterile sheath) to assist cannulation of the right internal jugular vein using the same Seldinger wire technique	Cannulation of the right internal jugular vein was attempted using a Seldinger wire technique guided by traditional surface landmarks	Pediatric patients presenting for cardiac surgery
Ishii et al. 2013	Open-label- RCT	Japan	59	Radial artery	In real time, the arterial puncture was guided by a SonoSite 180 ultrasound imaging device with a 2- to 7-MHz linear array transducer. The artery was imaged in its short axis	The operator used the pulsation of the radial artery as a guide for the cannulation	Infants and small children weighing 3–20 kg scheduled to undergo elective cardiac surgery for congenital heart disease
Law et al. 2014	Open-label- RCT	USA	201	Femoral artery	Probe and GE US machines (GE Healthcare, Wauwatosa, WI, USA) were used for all US access. The inguinal ligament, FV, and FA were identified with the US; color Doppler imaging was performed and stored. With the US probe in a longitudinal direction, the needle was monitored as it passed through the tissue into the vessel	All patients received general anesthesia followed by sterile prep and drape using an eyehole dressing. Landmarks of the anterior superior iliac spine, pubic symphysis, and inguinal ligament were identified. If present, palpation of the femoral artery (FA) pulse was performed. All patients then received a small subcutaneous dose of lidocaine, which initiated a timer for both groups. For both groups, if FA and femoral vein (FV) access was The intent was to access the FV first. However, if FA access was inadvertently obtained first, the appropriate sheath was placed even if the attempt was made for the vein (and vice versa)	All the pediatric patients scheduled for routine cardiac catheterizations during this period were eligible for inclusion in the study
Min et al. 2019	Patient blinded RCT	South Korea	74	Radial artery	The radial artery was identified in the US group using a linear ultrasound transducer in the short-axis view. We used the least depth-of-field setting of 1.5 cm, which allows maximum magnification of depth settings. After checking the depth of a patient’s radial artery from the skin, a 24-gauge angiocatheter was inserted at an angle of approximately 458 to the wrist. While watching the ultrasound screen closely, the needle was advanced until a bright white dot of the needle tip was observed above the radial artery	Radial arterial catheterization was performed using palpation of the radial arterial pulse. After the needle was inserted into the radial artery, the procedures were the same as those in group US, except ultrasonography	Infant patients under 12 months of age who were scheduled for elective cardiac Surgery due to congenital heart disease was enrolled in This study
Sadeghi et al. 2022	Open-label- RCT	Iran	120	Subclavian vein, IJV	Sonography was performed before cannulation in the US-guided group to assess the vein	Placement was performed via the Seldinger method. The finder syringe was entered at the vertex of the triangle between the sternocleidomastoid heads and the clavicle bone. Aspiration was performed with the finder syringe on the nipple side to detect the internal jugular vein; then, the central catheter and syringe were entered. A special CVP wire was entered into the primary syringe, and electrocardiographic monitoring was performed for arrhythmia occurrence. After the wire was positioned, the primary syringe was withdrawn, and the dilator was entered into the guide wire and fixed with a silk suture	The inclusion criteria were patients between 3 months and six years old undergoing cardiac surgeries
Salık et al. 2023	Open-label- RCT	Turkey	40	Femoral artery	A linear probe (5–12 MHz, Esaote, MyLab Six, the Netherlands) was employed in the US group of patients. The femoral artery and vein were spotted after the transducer was placed in a sterile sheath. Immediately distal of the inguinal ligament, the linear probe was placed on the short axis above the femoral artery. The femoral artery and vein were identified, and the femoral artery was confirmed using color Doppler ultrasonography when needed	The pulse of the femoral artery immediately distal of the inguinal ligament was determined manually in the patients of the palpation group. The 20-Ga needle was advanced until the artery puncture was performed. After achieving sufficient arterial blood flow, the guide wire (0.43 mm in size and 200 mm in length) was placed in the vessel's lumen. The catheter was sent over the guide wire, the cannula was placed using the Seldinger technique, and the guide wire was removed. After ensuring fixation with the suture, the access was completed	The study included neonatal patients with ASA scores of 3–4 who underwent congenital heart surgery between January 1, 2020 and January 1, 2021
Siddik-Sayyid et al. 2016	Open-label- RCT	Lebanon	106	Femoral artery	Patients in the ultrasound group had their femoral lines inserted under ultrasound guidance. The ultrasound equipment used was a SonoSite M-Turbo with an L25/13- to 6-MHz linear array transducer (SonoSite, Inc., Bothell, WA, USA). A sterile sheath covered the transducer. The inguinal area was scanned immediately distal to the inguinal ligament, and the femoral artery was identified. Using a short axis and an out-of-plane technique, the artery was centered in the middle of the screen, and the probe was held with the left-hand perpendicular to the artery	Patients in the palpation group had their femoral lines inserted using the palpation technique. The metallic cannula was inserted after localization of the femoral artery by identifying the pulse in the femoral triangle immediately distal to the inguinal ligament. The attempt was repeated until adequate arterial flow was obtained, which allowed the guidewire insertion. The metallic cannula was replaced with the catheter over the guidewire	Children under 12 years of age, ASA III or IV with congenital heart disease undergoing cardiac surgery
Verghese et al. 1999	Open label- RCT	USA	95	IJV	The Site Rite scanner (Dymax Corp., Pittsburgh, PA) was used in infants randomized for cannulation using ultrasound. This computerized unit is a portable, real-time, high-resolution imaging system designed to view the IJV and the CA. The probe uses a frequency of 9 MHz and a sector angle of 25", with the focal length positioned 1.5 cm from the cap. The image is displayed on a 7.62-cm diagonal display monitor. The 9-MHz transducer was covered by an elongated sterile sheath containing Aquasonic 100 (ultrasound transmission gel; Parker Laboratories, Inc, Fairfield, NJ) to maintain a sterile field	The traditional approach entailed identifying the external landmarks (sternocleidomastoid muscle, clavicle, sternal notch, and cricoid ring) and palpating the CA pulse. At the level of the cricoid ring and the apex of the triangle formed by the division of the sternocleidomastoid muscle and the base of the clavicle, a 21-gauge, 4-cm-long needle was inserted at a 30" angle lateral to the CA and directed toward the ipsilateral nipple. This point lies approximately lateral to the intersection of the CA with a line between the mastoid process and the suprasternal notch. The anesthesiologists used their left index and middle fingers to retract the pulsating CA. They identified the IJV puncture by the easy aspiration of dark venous blood from the vein through the needle. Using a standard Seldinger technique, a guide wire (0.018 inches in diameter, 40 cm long) was passed through the needle, followed by tissue dilation with a 5-French 8-cm-long dilator and advancement of a heparin-coated polyurethane 4-French (1%gauge), 8-cm (31/8 inch) double-lumen catheter (Cook Central Venous Catheter; Cook Critical Care, Bloomington, IN)	Ninety-five infants are scheduled for cardiovascular Surgery was studied in a prospective, randomized manner All the patients were younger than 12 months and Weighed less than 10 kg
Verghese et al. 2000	Open label- RCT	USA	45	IJV	The third technique used ultrasound imaging. The device used a 7.5 MHz transducer (SITERITE) and a two-dimensional image display. The carotid artery and right internal jugular vein could be visually distinguished by their relative position, the vein's compressibility, and the artery's significant pulsation at the level of the thyroid cartilage	This approach utilized visualization and palpation of the external landmarks, namely the carotid artery, sternocleidomastoid muscle, the clavicle, the sternal notch, and the cricoid cartilage. At the level of the cricoid (Y), the carotid artery was palpated, and the needle was inserted just lateral to the carotid at the apex of the triangle formed by the two divisions of the sternocleidomastoid muscle and the base of the clavicle. This site (X) lies roughly lateral to the intersection of the carotid artery (C ± D) with a line between the mastoid process and the suprasternal notch (A ± B). At this entry point, a 21-G 4.0 cm long needle with a syringe attached was directed towards the ipsilateral nipple. The pulsating carotid artery was retracted gently medially by the left hand's index and middle fingers	Infants aged one day to 12 months, ASA status III, weighing less than 10 kg, who were scheduled for cardiovascular surgery

**Table 2:** Baseline characters of the included population

Study	Number of patients in each group		Age (months), mean (SD)		Weight (kg), mean (SD)		Height (cm), mean (SD)		Gender (male), N (%)		Baseline heart rate, mean (SD)	
	U/S group	Palpation group	U/S group	Palpation group	U/S group	Palpation group	U/S group	Palpation group	U/S group	Palpation group	U/S group	Palpation group
Aktiz-Bıçak et al. 2023 (Femoral)	20	20	16.6 ± 10.0	14.6 ± 12.6	4.8 ± 2.2	5.7 ± 2.0	57.7 ± 10.3	63.4 ± 10.8	15 (75)	14 (70)	129 ± 11	120 ± 17
Aktiz-Bıçak et al. 2023 (IJV)	26	26	13.8 ± 14.6	15.6 ± 17.3	7.3 ± 2.9	7.9 ± 3.9	69.8 ± 11.6	71.7 ± 13.1	16 (61.5)	14 (53.8)	124 ± 12	123 ± 14
Alderson et al. 1993	20	20	8.5 ± 5.6	9.2 ± 7.2	6.6 ± 2.5	6.8 ± 2.5	N/A	N/A	N/A	N/A	N/A	N/A
Aouad et al. 2010	24	24	39.7 ± 30.8	29.8 ± 28.3	14.3 ± 7.2	11 ± 5.5	92.2 ± 19.8	82 ± 20.5	11 (45.8)	12 (50)	N/A	N/A
Grebenik et al. 2004	59	65	(1 day to 8 years)	(2 days to 7 years)	8.6 ± 5.4	8.9 ± 6.0	N/A	N/A	N/A	N/A	N/A	N/A
Ishii et al. 2013	59	59	17.8 ± 16.0	17.8 ± 16.1	8.3 ± 3.4	8.3 ± 3.5	N/A	N/A	N/A	N/A	N/A	N/A
Law et al. 2014	100	101	73.2 ± 124.8	97.2 ± 168.0	22.1 ± 26.1	26.2 ± 30	NA	NA	40 (40)	51 (51)	NA	NA
Min et al. 2019	37	37	1.7 ± 2.7	3.5 ± 3.5	4.8 ± 1.9	5.7 ± 2.1	56.0 ± 7.8	60.5 ± 8.1	18 (49)	24 (65)	153 ± 13	146 ± 17
Sadeghi et al. 2022	60	60	30.3 ± 25.9	30.3 ± 22.5	16.4 ± 24.6	11.4 ± 4.8	74.4 ± 26.5	87.4 ± 19.0	30 (50)	27 (45)	NA	NA
Salık et al. 2023	20	20	0.7 ± 0.4	0.6 ± 0.3	3.5 ± 0.4	3.4 ± 0.4	51.4 ± 1.9	51.2 ± 2.7	14 (70)	15 (70)	133 ± 15.1	130 ± 14.5
Siddik-Sayyid et al. 2016	53	53	37.9 ± 40.4	30.6 ± 25.7	10.6 ± 4.8	12.4 ± 8.0	82.7 ± 18.2	86.3 ± 26.1	33 (62.3)	29 (54.7)	116 ± 20.4	116 ± 20.6
Verghese et al. 1999	43	52	6.4 ± 3.8	5.9 ± 4.4	6.0 ± 2.3	5.8 ± 2.0	NA	NA	NA	NA	NA	NA
Verghese et al. 2000	16	16	8.0 ± 5.9	5.4 ± 4.1	6.0 ± 1.8	6.4 ± 2.3	NA	NA	NA	NA	NA	NA

Study	Baseline SBP (mmHg), mean (SD)		Baseline DBP (mmHg), mean (SD)		Baseline CVP (mmHg), mean (SD)		Vessel diameter (mm), mean (SD)		Previous cannulation, N (%)	
	U/S group	Palpation group	U/S group	Palpation group	U/S group	Palpation group	U/S group	Palpation group	U/S group	Palpation group
Aktiz-Bıçak et al. 2023 (Femoral)	69 ± 21	81 ± 19	40 ± 16	44 ± 12	4.7 ± 2.8	3.9 ± 3.2	2.0 ± 0.3	2.2 ± 0.4	13 (65)	16 (80)
Aktiz-Bıçak et al. 2023 (IJV)	87 ± 14	82 ± 14	50 ± 10	46 ± 8	4.6 ± 3.5	4.3 ± 3.0	5.7 ± 1.2	6.1 ± 0.9	6 (23.1)	6 (23.1)
Alderson et al. 1993	N/A	N/A	N/A	N/A	N/A	N/A	N/A	N/A	0 (0)	0 (0)
Aouad et al. 2010	N/A	N/A	N/A	N/A	N/A	N/A	N/A	N/A	N/A	N/A
Grebenik et al. 2004	N/A	N/A	N/A	N/A	N/A	N/A	N/A	N/A	N/A	N/A
Ishii et al. 2013	N/A	N/A	N/A	N/A	N/A	N/A	N/A	N/A	N/A	N/A
Law et al. 2014	NA	NA	NA	NA	NA	NA	NA	NA	39 (39)	43 (42.6)
Min et al. 2019	57 ± 16	62 ± 17	NA	NA	NA	NA	1.3 ± 0.6	1.3 ± 0.4	NA	NA
Sadeghi et al. 2022	NA	NA	NA	NA	NA	NA	NA	NA	39 (65)	43 (71.7)
Salık et al. 2023	57.9 ± 11.3	65.3 ± 17.0	32.2 ± 9.6	35.7 ± 12.9	4.7 ± 2.7	4.7 ± 2.7	1.9 ± 0.2	1.9 ± 0.2	13 (65)	16 (80)
Siddik-Sayyid et al. 2016	94.3 ± 14.4	94.3 ± 17.7	57 ± 12.4	57 ± 12.4	NA	NA	3.2 ± 1.2	3.3 ± 1.1	NA	NA
Verghese et al. 1999	NA	NA	NA	NA	NA	NA	NA	NA	NA	NA
Verghese et al. 2000	NA	NA	NA	NA	NA	NA	NA	NA	NA	NA

### Risk of Bias and Certainty of Evidence

**Fig. 2:**
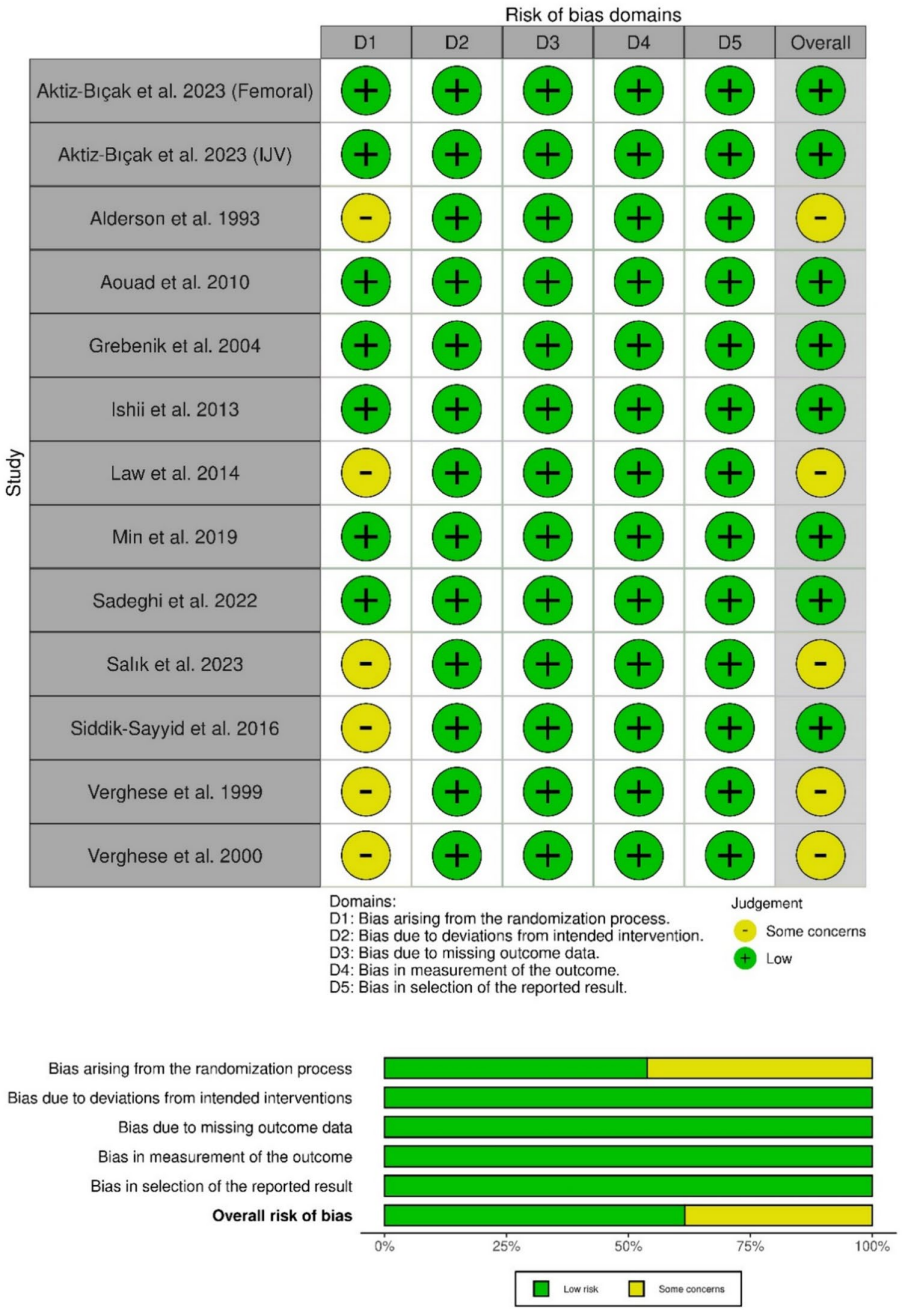
Quality assessment of risk of bias in the included trials. The upper panel presents a schematic representation of risks (low = green, unclear = yellow, and high = red) for specific types of biases of each of the studies in the review. The lower panel presents risks (low = green, unclear = yellow, and high = red) for the subtypes of biases of the combination of studies included in this review

Among the 13 studies reviewed, eight were categorized as having low risk regarding the five domains [15, 22, 23, 25, 26, 28, 29], whereas the remaining studies were assessed to have some concern of selection bias [24, 27, 30–33]. The risk of bias for each of the included studies is shown in Fig. 2. Certainty of evidence is demonstrated in a GRADE evidence profile (Table 3).

**Table 3:** GRADE certainty of evidence assessment

Certainty assessment						
Participants (studies) follow-up	Risk of bias	Inconsistency	Indirectness	Imprecision	Publication bias	Overall certainty of evidence
*Successful arterial cannulation*						
579 (6 RCTs)	Not serious	Very serious^a^	Not serious	Serious^b^	None	⨁◯◯◯ Very low
*Successful venous cannulation*						
391 (6 RCTs)	Not serious	Very serious^a^	Not serious	Serious^b^	None	⨁◯◯◯ Very low
*First attempt success (arterial)*						
579 (6 RCTs)	Not serious	Very serious^a^	Not serious	Not serious	None	⨁⨁◯◯ Low
*First attempt success (venous)*						
220 (3 RCTs)	Not serious	Very serious^a^	Not serious	Very serious^c^	None	⨁◯◯◯ Very low
*Number of attempts (arterial)*						
579 (6 RCTs)	Not serious	Serious^d^	Not serious	Not serious	None	⨁⨁⨁◯ Moderate
*Number of attempts (venous)*						
235 (4 RCTs)	Not serious	Very serious^a^	Not serious	Serious^b^	None	⨁◯◯◯ Very low
*Time of attempted cannulation (arterial)*						
461 (5 RCTs)	Not serious	Not serious	Not serious	Not serious	None	⨁⨁⨁⨁ High
*Time of attempted cannulation (venous)*						
347 (5 RCTs)	Not serious	Very serious^a^	Not serious	Very serious^b^	None	⨁◯◯◯ Very low
*Any complications (arterial)*						
356 (5 RCTs)	Not serious	Not serious	Not serious	Serious^e^	None	⨁⨁⨁◯ Moderate
*Any complications (venous)*						
463 (6 RCTs)	Not serious	Serious^d^	Not serious	Very serious^c^	NONE	⨁◯◯◯ Very low

^a^I2 > 75%, ^b^a wide confidence interval that does not exclude the risk of appreciable harm/benefit, ^c^a wide confidence interval that does not exclude the risk of appreciable harm/benefit, with a low number of events, ^d^I2 > 50%, ^e^a low number of events

### Primary Outcomes: Successful Cannulation

**Fig. 3:**
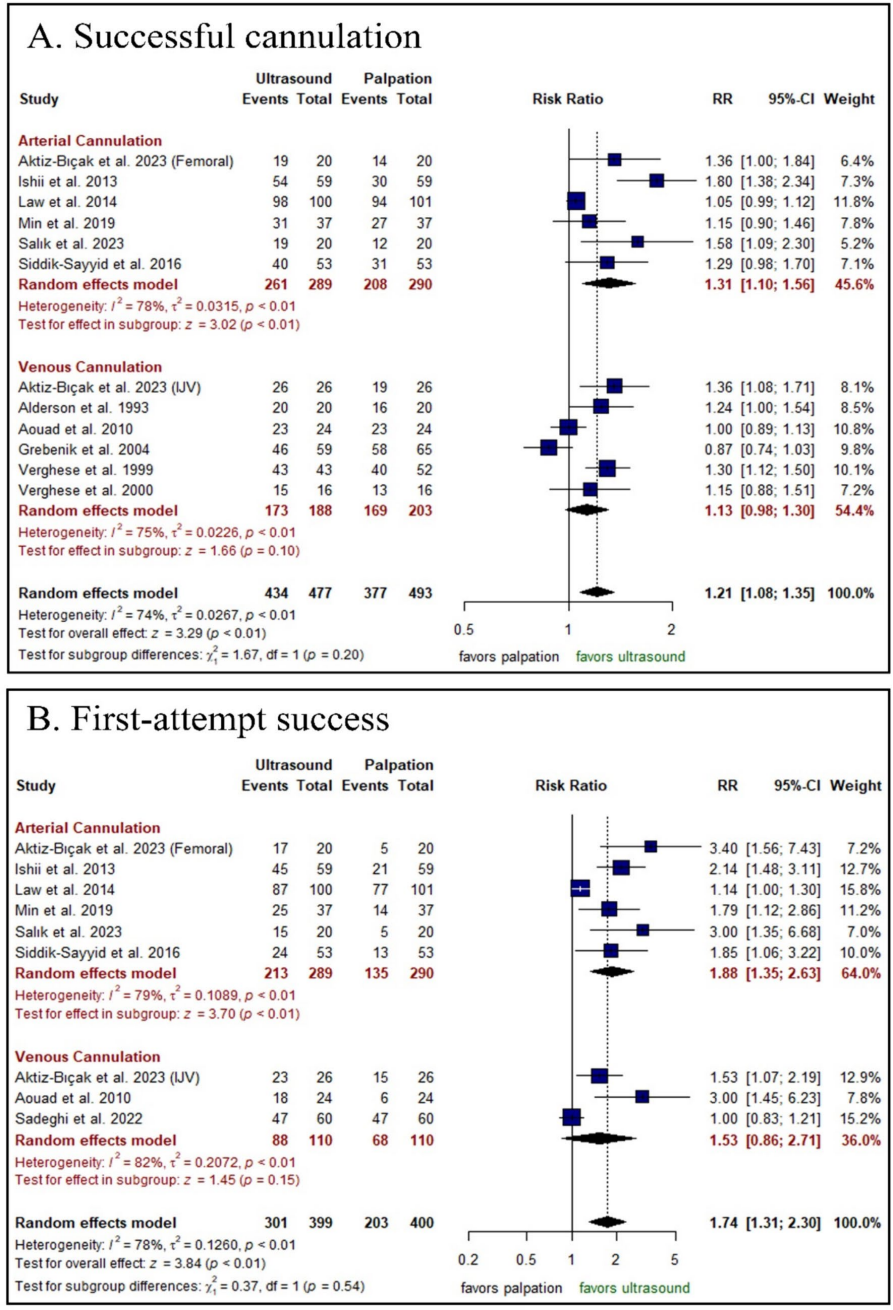
Forest plot of the primary efficacy outcomes, *RR* risk ratio, *CI* confidence interval

The US-guided technique significantly increased the successful cannulation in arterial cannulation [RR: 1.31 with 95% CI (1.10, 1.56), P < 0.0001]; however, there was no significant difference between both groups in venous cannulation [RR: 1.13 with 95% CI (0.98, 1.30), P = 0.10] (Fig. 3A). Moreover, the US-guided technique significantly increased the first-attempt success in arterial cannulation [RR: 1.88 with 95% CI (1.35, 2.63), P < 0.0001]; however, there was no significant difference between the two groups in venous cannulation [RR: 1.53 with 95% CI (0.86, 2.71), P = 0.15] (Fig. 3B).

Pooled studies were heterogeneous in both outcomes (P < 0.001, I2 = 74%) and (P < 0.001, I2 = 78%), respectively. Sensitivity analysis was not applicable in both outcomes (Figs. S1–S4). We performed a meta-regression analysis against successful cannulation based on known baseline characteristics such as age (m) and weight (kg), with no obvious impact on the effect size (Table S2). A funnel plot was used in successful cannulation to detect possible publication bias. We found significant asymmetry by inspection, indicating significant publication bias (Egger’s P value = 0.029) (Fig. S5). Moreover, the trim and fill method was employed to address this, as shown in Fig. S6. Finally, the test of subgroup analysis was insignificant in successful cannulation either based on the vessel cannulated (arterial vs. venous) or the artery cannulated (femoral vs. radial) (P = 0.20), (P = 0.93) respectively (Fig. S7), and in first-attempt success based on the vessel cannulated (arterial vs. venous) (P = 0.54).

### Secondary Outcomes

#### Efficacy Outcomes

**Fig. 4:**
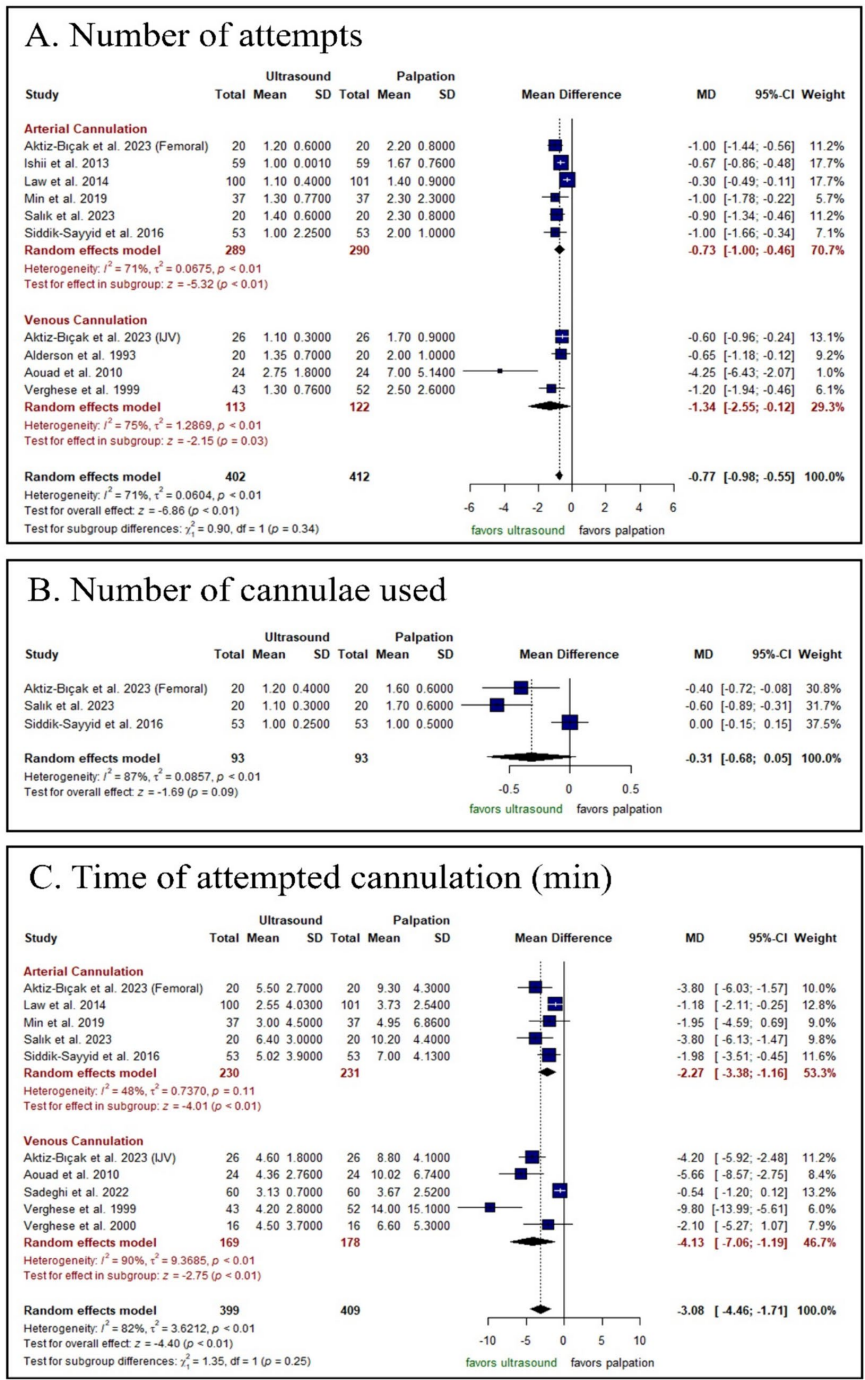
Forest plot of the secondary efficacy outcomes, *RR* risk ratio, *CI* confidence interval

The US-guided technique significantly decreased the number of attempts either in arterial cannulation [MD: − 0.73 with 95% CI (− 1.00, − 0.46), P < 0.0001] or in venous cannulation [MD: − 1.34 with 95% CI (− 2.55, − 0.12), P = 0.03] (Fig. 4A). However, there was no significant difference between both groups in arterial cannulation regarding the number of cannulas used [RR: − 0.31 with 95% CI (− 0.68, 0.05), P = 0.09] (Fig. 4B). Pooled studies were heterogeneous in both outcomes (P < 0.001, I2 = 71%) and (P < 0.001, I2 = 87%), respectively.

Regarding the number of attempts, heterogeneity was best resolved after omitting the study by Law et al. (2014) (I2 = 43%) (Aouad et al. (2010) and Law et al. (2014) were detected as possible outliers [random‐effect model]) (Figs. S8–S9). Results with these outliers removed are shown in Fig. S10. Moreover, in the number of cannulas used, sensitivity analysis revealed that the heterogeneity was best resolved after omitting the study by Siddik-Sayyid et al. (2016) (I2 = 0%) (no outliers detected [random effect model]) (Figs. S11–S12).

We performed a meta-regression analysis against the number of attempts based on known baseline characteristics such as age (m) and weight (kg), with a noticeable impact on the effect size with [P < 0.0001], [P < 0.0001] respectively (Table S2). A bubble plot of meta-regression is shown in Figs. S13–S14.

By inspection of funnel plot, we found significant asymmetry, indicating that there was significant publication bias (Egger’s P value = 0.005) (Fig. S15); the trim and fill method was employed to address this as shown in Fig. S16. Finally, the subgroup analysis test was insignificant in the number of attempts, either based on the vessel cannulated (arterial vs. venous) or the artery cannulated (femoral vs. radial) (P = 0.34), and (P = 0.80), respectively (Fig. S17).

#### Procedural Outcome: Time of Attempted Cannulation

The US-guided technique was associated with a significantly decreased the time of attempted cannulation either in arterial cannulation [MD: − 2.27 with 95% CI (− 3.38, − 1.16), P < 0.0001] or in venous cannulation [MD: − 4.13 with 95% CI (− 7.06, − 1.19), P < 0.0001] (Fig. 4C). Pooled studies were heterogeneous (P < 0.001, I2 = 82%). Sensitivity analysis was not applicable (Sadeghi et al. [29] and Verghese et al. [32] were detected as possible outliers [random effect model]) (Figs. S18–S19). Results with these outliers removed are shown in Fig. S20.

We performed a meta-regression analysis against the time of attempted cannulation based on known baseline characteristics such as age (m) and weight (kg), with no obvious impact on the effect size (Table S2). By inspection of funnel plot, we found significant asymmetry, indicating that there was significant publication bias (Egger’s P value = 0.001) (Fig. S21); moreover, trim and fill method was employed to address this as shown in Fig. S22. Finally, the subgroup analysis test was insignificant in the number of attempts based on the vessel cannulated (arterial vs. venous) or the artery cannulated (femoral vs. radial) (P = 0.25) and (P = 0.77), respectively (Fig. S23).

#### Safety Outcomes

##### Failure Causes

The US-guided technique significantly decreased the incidence of failure to pass a guide wire either in arterial cannulation [RR: − 0.20 with 95% CI (0.05, 0.86), P = 0.03] or in venous cannulation [RR: 0.24 with 95% CI (0.08, 0.71), P < 0.001] (Fig. S24). However, there was no significant difference between both groups in arterial cannulation regarding the incidence of failure to puncture the vessel with [RR: 0.20 with 95% CI (0.01, 3.91), P = 0.31] (Fig. S25). Pooled studies were heterogeneous in both outcomes with (P = 0.74, I2 = 0%), (P = 1.00, I2 = 0%) respectively.

##### Complications

**Fig. 5:**
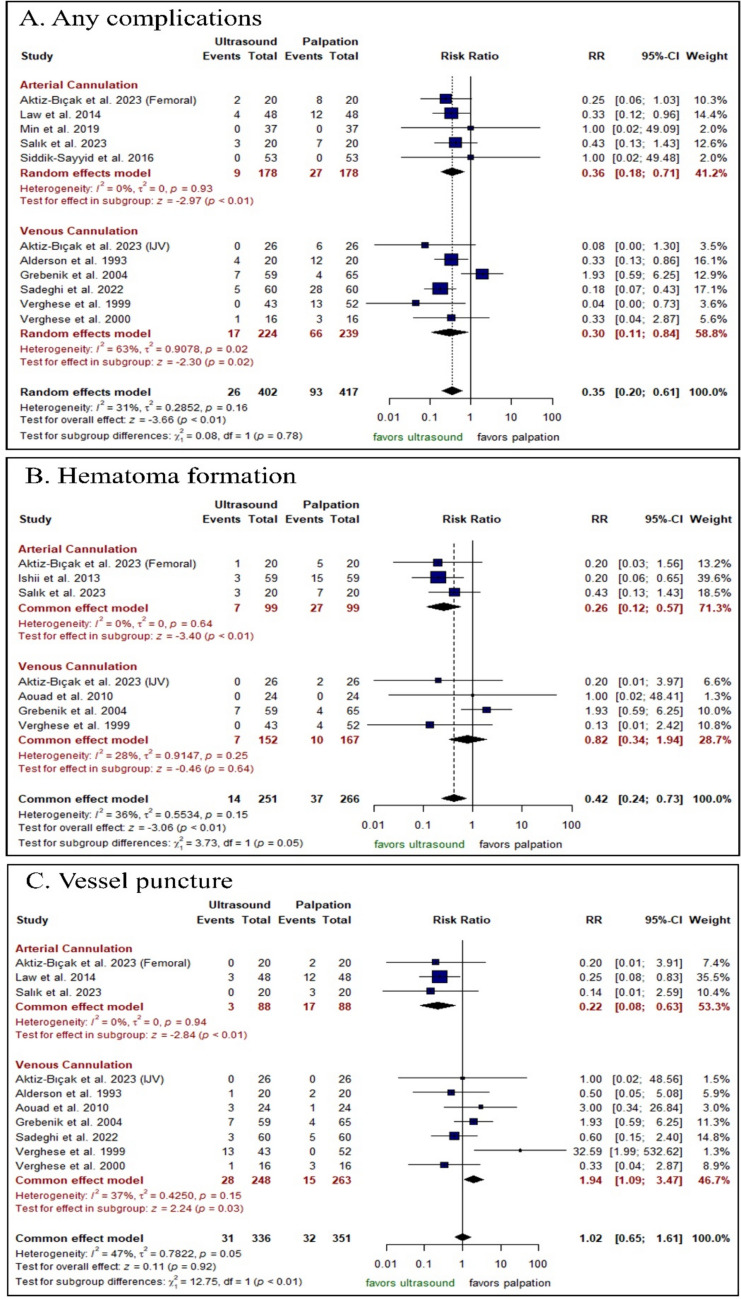
Forest plot of the safety outcomes, *RR* risk ratio, *CI* confidence interval

The US-guided technique significantly decreased the incidence of any complications either in arterial cannulation [RR: 0.36 with 95% CI (0.18, 0.71), P < 0.001] or in venous cannulation [RR: 0.30 with 95% CI (0.11, 0.84), P = 0.02] (Fig. 5A). Furthermore, the US-guided technique significantly decreased in the incidence of hematoma formation in arterial cannulation [RR: 0.26 with 95% CI (0.12, 0.57), P < 0.001]; however, there was no significant difference between both groups in venous cannulation [RR: 0.82 with 95% CI (0.34, 1.94), P = 0.64] (Fig. 5B). Moreover, the US-guided technique significantly decreased the incidence of vessel puncture in arterial cannulation [RR: 0.22 with 95% CI (0.08, 0.63), P < 0.001]; however, it significantly increased the incidence of vessel puncture in venous cannulation with [RR: 1.94 with 95% CI (1.09, 3.47), P = 0.03] (Fig. 5C). Moreover, there was no significant difference between both groups in surgical cutdown in arterial cannulation [RR: 0.43 with 95% CI (0.07, 2.75), P = 0.37] (Fig. S26).

Pooled studies were homogenous in any complications (P = 0.31, I2 = 16%), hematoma formation (P = 0.15, I2 = 36%), vessel puncture (P = 0.05, I2 = 47%), and in surgical cutdown (P = 0.84, I2 = 0%). Regarding any complication in venous cannulation (I2 = 63%), sensitivity analysis revealed that the heterogeneity was best resolved after omitting the study by Grebenik et al. (2004) (I2 = 0%) (no outliers detected [random effect model]) (Fig. S27).

By inspection of funnel plot of vessel puncture, we did not find significant asymmetry, indicating that there was no significant publication bias (Egger’s P value = 0.88) and (Egger’s P value = 0.80), respectively (Figs. S28–S29). Finally, the test of subgroup analysis was not significant across all the outcomes (P > 0.1) (Figs. S30–S31), except for vessel puncture, the test of subgroup analysis was significant based on the vessel cannulated (arterial vs. venous) (P < 0.001) with arterial cannulation being associated with a lower incidence of vessel puncture [RR: 0.22 with 95% CI (0.08, 0.63), P < 0.001] compared to venous cannulation [RR: 1.94 with 95% CI (1.09, 3.47), P = 0.03].

## Discussion

Our meta-analysis on ultrasound-guided versus palpation-guided techniques for vascular access in pediatric cardiac surgery revealed that ultrasound-guided technique significantly increases the incidence of successful cannulation and first-attempt success compared to palpation-guided methods. Additionally, ultrasound guidance significantly reduced the number of attempts and procedural time required for arterial and venous cannulation. Furthermore, it was associated with decreased complications and procedure failure. Despite these advantages, ultrasound-guided venous cannulation was associated with a higher incidence of vessel puncture.

Our findings align with several studies that have reported similar results, indicating the superiority of ultrasound guidance in enhancing vascular access success rates [34–39]. Additionally, a meta-analysis on adult populations undergoing central venous catheterization has consistently shown improved success rates and reduced complication rates with ultrasound-guided techniques compared to palpation-guided methods [40]. Furthermore, an updated meta-analysis conducted by Gao et al. [41] and included both adults and pediatric populations demonstrated the benefit of the ultrasound-guided technique over the palpation technique. The extrapolation of these findings to pediatric populations, particularly in the context of cardiac surgery, underscores the generalized applicability of ultrasound guidance in optimizing procedural outcomes.

The suggested mechanisms underlying the superior success rates associated with ultrasound-guided techniques in the literature can be attributed to ultrasound providing real-time visualization of vascular structures, enabling clinicians to accurately identify vessel anatomy, size, and depth, thereby facilitating precise needle placement. This direct visualization minimizes the risk of inadvertent punctures, reducing procedural complications and optimizing cannulation success [42, 43].

The number and time of attempts during vascular access procedures are crucial metrics that reflect procedural efficiency. Our findings indicate that ultrasound-guided techniques significantly reduce both the number of attempts and the time required for vascular access compared to palpation-guided methods. This aligns with previous, where ultrasound guidance has consistently demonstrated advantages in procedural efficiency and success rates [44]. Like successful canulation, decreased time and number of attempts in US-guided procedures could be attributed to real-time visualization of vascular structures, allowing precise needle placement and minimizing the need for multiple insertion attempts.

Regarding the incidence of failure and complications, our findings indicate that ultrasound-guided techniques are associated with a significant decrease in the incidence of failure and complications such as hematoma formation and vessel puncture, particularly in arterial cannulation. This aligns with previous meta-analyses demonstrating the benefits of ultrasound guidance in reducing the incidence of complications [44, 45]. In contrast to our findings regarding hematoma, the previous meta-analysis conducted by Pacha et al. [40] on the adult population demonstrated no significant difference between both techniques regarding the incidence of hematoma. Nevertheless, our study's subgroup analysis revealed differences based on factors such as the type of vessel cannulated, and the specific artery targeted. For example, ultrasound-guided venous cannulation was associated with a higher incidence of vessel puncture than palpation-guided methods, suggesting the need for cautious consideration of procedural techniques and anatomical factors.

### Strength and Limitations

Our study was the first meta-analysis conducted to compare US and palpation-guided techniques in vascular access in children undergoing cardiac surgery, encompassing a comprehensive literature search across multiple databases, ensuring the identification of relevant RCTs. The study further conducted subgroup analyses to explore outcome variations based on factors such as the type of vessel cannulated and the specific artery targeted, thus providing granularity to the analysis. Furthermore, sensitivity analyses and meta-regression were performed to assess the robustness of the findings and evaluate the impact of individual studies and baseline characteristics on the overall results, enhancing the reliability and validity of the meta-analysis. However, we faced some limitations. The included studies exhibited heterogeneity, which may have influenced the robustness of the findings despite attempts to account for heterogeneity through subgroup and sensitivity analyses. Additionally, publication bias cannot be ruled out, as studies with positive results may be more likely to be published, potentially leading to overestimating the effect sizes associated with ultrasound-guided techniques.

### Clinical Implications

Our results underscore the importance of incorporating ultrasound guidance into clinical practice to improve procedural efficiency and patient outcomes. To maximize the benefits of this approach in pediatric cardiac surgery settings, healthcare providers should consider adopting standardized protocols for ultrasound-guided techniques and ensuring adequate training.

## Conclusion

Ultrasound guidance improves successful cannulation rates and first-attempt success in arterial access while reducing the number of attempts and procedural time for both arterial and venous access. It was also associated with a lower incidence of complications and procedure failure, particularly in the arterial setting. However, it was associated with a higher incidence of venous puncture.

## Supplementary Information

Below is the link to the electronic supplementary material.

Supplementary file1 (DOCX 569 KB)

## Data Availability

Not applicable.
