# Supplementary material for: Ultrasound-Guided Versus Palpation-Guided Techniques to Achieve Vascular Access in Children Undergoing Cardiac Surgery: A Systematic Review and Meta-analysis of Randomized Controlled Trials
Source: Pediatr Cardiol. 2024 Jul 27;46(6):1426–47. doi: 10.1007/s00246-024-03581-y (PMC12296815; doi:10.1007/s00246-024-03581-y)
Supplement: Supplementary file 1 — Supplementary file1 (DOCX 569 KB) [file 246_2024_3581_MOESM1_ESM.docx]

**Supplementary material:**

**Title.**

**Ultrasound-guided versus palpation-guided techniques to achieve vascular access in children undergoing cardiac surgery. A systematic review and meta-analysis of randomized controlled trials.**

**Running Title.**

US for pediatrics’ vascular access.

**Authors.**

Ahmed A. Ibrahim^1*^, Abdallah R. Allam^1*^, Ahmed Mazen Amin^2^, Mohamed Saad Rakab^2^, Abdulhameed Alhadeethi^3^, Ahmed W. Hageen^4^, Abdelrahman Mahmoud^5^, Mohamed Abuelazm^4^, Basel Abdelazeem^6^.

**Affiliations.**

1. Faculty of Medicine, Menoufia University, Menoufia, Egypt.
2. Faculty of Medicine, Mansoura University, Mansoura, Egypt.
3. Department of General Medicine, Al-Salam Teaching Hospital, Ninevah, Iraq.
4. Faculty of Medicine, Tanta University, Tanta, Egypt.
5. Faculty of Medicine, Minia University, Minia, Egypt.
6. Department of Cardiology, West Virginia University, Morgantown, West Virginia, USA.

*, Both authors have equal contributions and are co-first authors.

**Keywords**.

Cardiac surgery; pediatrics; ultrasound; cannula; catheter; review.

**Corresponding author.**

**Ahmed A. Ibrahim, MBBCh.**

Address: Saad Zaghloul St., Shibin El-Kom, Menoufia Governorate, Egypt, 32511.

Tel: +201223131497.

Email: [Ahmedabdalaziz33@med.menofia.edu.eg](mailto:Ahmedabdalaziz33@med.menofia.edu.eg); [Drahmedabdelaziz99@gmail.com](mailto:Drahmedabdelaziz99@gmail.com).

ORCID: 0000-0003-3669-5724.

**Contents.**

**Tables:**

Table S1: Search strategy.

Table S2: Meta-regression analysis based on age, and weight.

Table S3: The PRISMA checklist.

**Figures:**

Figure S1: Sensitivity analysis of successful cannulation.

Figure S2: Baujat plot of successful cannulation (The x-axis of the Baujat plot shows the overall heterogeneity contribution of each effect size while the y-axis shows the influence of each effect size on the pooled result).

Figure S3: Sensitivity analysis of first-attempt success.

Figure S4: Baujat plot of first-attempt success (The x-axis of the Baujat plot shows the overall heterogeneity contribution of each effect size while the y-axis shows the influence of each effect size on the pooled result).

Figure S5: Funnel plot of successful cannulation.

Figure S6: Trim and fill plot of successful cannulation.

Figure S7: Subgroup analysis of successful cannulation based on the artery cannulated (Femoral Vs. Radial).

Figure S8: Sensitivity analysis of number of attempts.

Figure S9: Baujat plot of number of attempts (The x-axis of the Baujat plot shows the overall heterogeneity contribution of each effect size while the y-axis shows the influence of each effect size on the pooled result).

Figure S10: Results of number of attempts with outliers removed.

Figure S11: Sensitivity analysis of number of cannulae used.

Figure S12: Baujat plot of cannulae used (The x-axis of the Baujat plot shows the overall heterogeneity contribution of each effect size while the y-axis shows the influence of each effect size on the pooled result).

Figure S13: Bubble plot of meta-regression analysis of number of attempts based on age.

Figure S14: Bubble plot of meta-regression analysis of number of attempts based on weight.

Figure S15: Funnel plot of number of attempts.

Figure S16: Trim and fill plot of number of attempts.

Figure S17: Subgroup analysis of number of attempts based on the artery cannulated (Femoral Vs. Radial).

Figure S18: Sensitivity analysis of number of the time of attempted cannulation.

Figure S19: Baujat plot of the time of attempted cannulation (The x-axis of the Baujat plot shows the overall heterogeneity contribution of each effect size while the y-axis shows the influence of each effect size on the pooled result).

Figure S20: Results of the time of attempted cannulation with outliers removed.

Figure S21: Funnel plot of time of attempted cannulation.

Figure S22: Trim and fill plot of time of attempted cannulation.

Figure S23: Subgroup analysis of time of attempted cannulation based on the artery cannulated (Femoral Vs. Radial).

Figure S24: Forest plot of failure to pass a guide wire.

Figure S25: Forest plot of failure to puncture the vessel.

Figure S26: Forest plot of surgical cutdown.

Figure S27: Sensitivity analysis of number of any complication in venous cannulation.

Figure S28: Funnel plot of any complication.

Figure S29: Funnel plot of vessel puncture.

Figure S30: Subgroup analysis of any complication based on the artery cannulated (Femoral Vs. Radial).

Figure S31: Subgroup analysis of hematoma formation based on the artery cannulated (Femoral Vs. Radial).

Table S1: Search strategy.

| **Database** | **Search Terms** | **Search Field** | **Search Results** |
| --- | --- | --- | --- |
| **PubMed** | (ultraso* OR Ultrasonograph* OR Echotomograph* OR sonograph*) AND (palpation OR landmark) AND ("cardiac surgery" OR "heart surgery") | All field | 58 |
| **Cochrane** | (ultraso* OR Ultrasonograph* OR Echotomograph* OR sonograph*) AND (palpation OR landmark) AND ("cardiac surgery" OR "heart surgery") | All field | 44 |
| **WOS** | (ultraso* OR Ultrasonograph* OR Echotomograph* OR sonograph*) AND (palpation OR landmark) AND ("cardiac surgery" OR "heart surgery") | All field | 61 |
| **SCOPUS** | (ultraso* OR Ultrasonograph* OR Echotomograph* OR sonograph*) AND (palpation OR landmark) AND ("cardiac surgery" OR "heart surgery") | Title, Abstract, Keywords | 76 |
| **EMBASE** | #4.  #1 AND #2 AND #3                                            56  #3.  'cardiac surgery':ti,ab,kw OR 'heart                    98,509       surgery':ti,ab,kw  #2.  palpation:ti,ab,kw OR landmark:ti,ab,kw                 54,065  #1.  ultraso*:ti,ab,kw OR ultrasonograph*:ti,ab,kw OR       753,223       echograph:ti,ab,kw OR echotomography:ti,ab,kw OR       sonograph*:ti,ab,kw | All field | 56 |

Table S2: Meta-regression analysis based on age, and weight.

| Variables | Slope point estimate (lower limit to upper limit) | P-value | Slope point estimate (lower limit to upper limit) | P-value | Slope point estimate (lower limit to upper limit) | P-value |
| --- | --- | --- | --- | --- | --- | --- |
|  | **Successful cannulation** |  | **Time of attempted cannulation (min)** |  | **Number of attempts** |  |
| **Age, m** | -0.001 (-0.006, 0.002) | 0.171 | 0.035 (-0.015, 0.085) | 0.176 | **0.0065 (0.0032, 0.0098)** | **<0.0001** |
| **Weight, kg** | -0.012 (-0.031, 0.005) | 0.181 | 0.161 (-0.033, 0.357) | 0.104 | **0.0275 (0.0136, 0.0414)** | **<0.0001** |

Table S3: The PRISMA checklist.

| **Section and Topic** | **Item #** | **Checklist item** | **Location where item is reported** |
| --- | --- | --- | --- |
| **TITLE** | | |  |
| Title | 1 | Identify the report as a systematic review. | Line 3 |
| **ABSTRACT** | | |  |
| Abstract | 2 | See the PRISMA 2020 for Abstracts checklist. | Page 3 |
| **INTRODUCTION** | | |  |
| Rationale | 3 | Describe the rationale for the review in the context of existing knowledge. | Pages 5-6 |
| Objectives | 4 | Provide an explicit statement of the objective(s) or question(s) the review addresses. | Page 6 |
| **METHODS** | | |  |
| Eligibility criteria | 5 | Specify the inclusion and exclusion criteria for the review and how studies were grouped for the syntheses. | Pages 7-8, subsection 2.3 |
| Information sources | 6 | Specify all databases, registers, websites, organisations, reference lists and other sources searched or consulted to identify studies. Specify the date when each source was last searched or consulted. | Page 7, subsection 2.2 |
| Search strategy | 7 | Present the full search strategies for all databases, registers and websites, including any filters and limits used. | Supplementary material, table S2 |
| Selection process | 8 | Specify the methods used to decide whether a study met the inclusion criteria of the review, including how many reviewers screened each record and each report retrieved, whether they worked independently, and if applicable, details of automation tools used in the process. | Page 8, subsection 2.4 |
| Data collection process | 9 | Specify the methods used to collect data from reports, including how many reviewers collected data from each report, whether they worked independently, any processes for obtaining or confirming data from study investigators, and if applicable, details of automation tools used in the process. | Page 8, subsection 2.5 |
| Data items | 10a | List and define all outcomes for which data were sought. Specify whether all results that were compatible with each outcome domain in each study were sought (e.g. for all measures, time points, analyses), and if not, the methods used to decide which results to collect. | Page 8, subsection 2.5 |
|  | 10b | List and define all other variables for which data were sought (e.g. participant and intervention characteristics, funding sources). Describe any assumptions made about any missing or unclear information. | Page 8, subsection 2.5 |
| Study risk of bias assessment | 11 | Specify the methods used to assess risk of bias in the included studies, including details of the tool(s) used, how many reviewers assessed each study and whether they worked independently, and if applicable, details of automation tools used in the process. | Pages 8-9, subsection 2.6 |
| Effect measures | 12 | Specify for each outcome the effect measure(s) (e.g. risk ratio, mean difference) used in the synthesis or presentation of results. | Page 9, subsection 2.7 |
| Synthesis methods | 13a | Describe the processes used to decide which studies were eligible for each synthesis (e.g. tabulating the study intervention characteristics and comparing against the planned groups for each synthesis (item #5)). | Page 9, subsection 2.7 |
|  | 13b | Describe any methods required to prepare the data for presentation or synthesis, such as handling of missing summary statistics, or data conversions. | Page 9, subsection 2.7 |
|  | 13c | Describe any methods used to tabulate or visually display results of individual studies and syntheses. | Page 9, subsection 2.7 |
|  | 13d | Describe any methods used to synthesize results and provide a rationale for the choice(s). If meta-analysis was performed, describe the model(s), method(s) to identify the presence and extent of statistical heterogeneity, and software package(s) used. | Page 9, subsection 2.7 |
|  | 13e | Describe any methods used to explore possible causes of heterogeneity among study results (e.g. subgroup analysis, meta-regression). | Page 9, subsection 2.7 |
|  | 13f | Describe any sensitivity analyses conducted to assess robustness of the synthesized results. | Page 9, subsection 2.7 |
| Reporting bias assessment | 14 | Describe any methods used to assess risk of bias due to missing results in a synthesis (arising from reporting biases). | Pages 8-9, subsection 2.6 |
| Certainty assessment | 15 | Describe any methods used to assess certainty (or confidence) in the body of evidence for an outcome. | Pages 8-9, subsection 2.6 |
| **RESULTS** | | |  |
| Study selection | 16a | Describe the results of the search and selection process, from the number of records identified in the search to the number of studies included in the review, ideally using a flow diagram. | Pages 9-10, subsection 3.1 |
|  | 16b | Cite studies that might appear to meet the inclusion criteria, but which were excluded, and explain why they were excluded. | Not applicable |
| Study characteristics | 17 | Cite each included study and present its characteristics. | Page 10, subsection 3.2 |
| Risk of bias in studies | 18 | Present assessments of risk of bias for each included study. | Page 10, subsection 3.3 |
| Results of individual studies | 19 | For all outcomes, present, for each study: (a) summary statistics for each group (where appropriate) and (b) an effect estimates and its precision (e.g. confidence/credible interval), ideally using structured tables or plots. | Pages 10-14, subsections 3.4-3.5 |
| Results of syntheses | 20a | For each synthesis, briefly summarise the characteristics and risk of bias among contributing studies. | Page 10, subsection 3.3 |
|  | 20b | Present results of all statistical syntheses conducted. If meta-analysis was done, present for each the summary estimate and its precision (e.g. confidence/credible interval) and measures of statistical heterogeneity. If comparing groups, describe the direction of the effect. | Pages 10-14, subsections 3.4-3.5 |
|  | 20c | Present results of all investigations of possible causes of heterogeneity among study results. | Pages 10-14, subsections 3.4-3.5 |
|  | 20d | Present results of all sensitivity analyses conducted to assess the robustness of the synthesized results. | Pages 10-14, subsections 3.4-3.5 |
| Reporting biases | 21 | Present assessments of risk of bias due to missing results (arising from reporting biases) for each synthesis assessed. | Page 10, subsection 3.3 |
| Certainty of evidence | 22 | Present assessments of certainty (or confidence) in the body of evidence for each outcome assessed. | Table 3 |
| **DISCUSSION** | | |  |
| Discussion | 23a | Provide a general interpretation of the results in the context of other evidence. | Page 15 |
|  | 23b | Discuss any limitations of the evidence included in the review. | Pages 16-17 |
|  | 23c | Discuss any limitations of the review processes used. | Pages 16-17 |
|  | 23d | Discuss implications of the results for practice, policy, and future research. | Page 18 |
| **OTHER INFORMATION** | | |  |
| Registration and protocol | 24a | Provide registration information for the review, including register name and registration number, or state that the review was not registered. | Page 7, subsection 2.1 |
|  | 24b | Indicate where the review protocol can be accessed, or state that a protocol was not prepared. | Page 7, subsection 2.1 |
|  | 24c | Describe and explain any amendments to information provided at registration or in the protocol. | Page 7, subsection 2.1 |
| Support | 25 | Describe sources of financial or non-financial support for the review, and the role of the funders or sponsors in the review. | Page 18 |
| Competing interests | 26 | Declare any competing interests of review authors. | Page 18 |
| Availability of data, code and other materials | 27 | Report which of the following are publicly available and where they can be found template data collection forms; data extracted from included studies; data used for all analyses; analytic code; any other materials used in the review. | Page 18 |


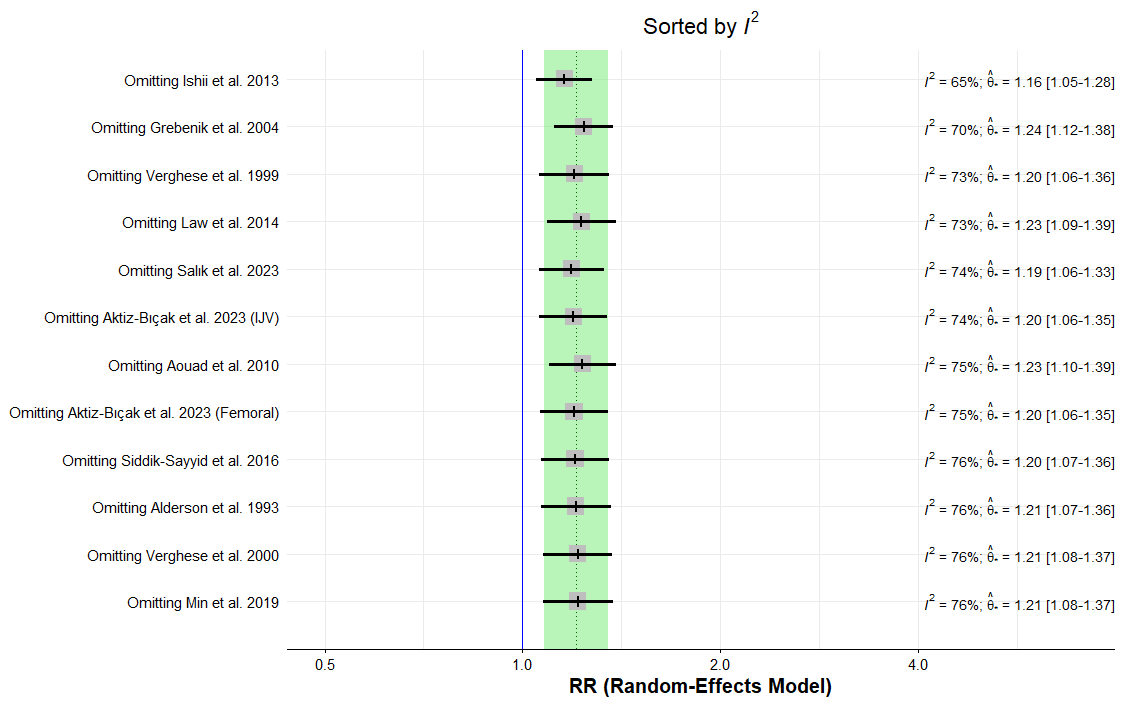


Figure S1: Sensitivity analysis of successful cannulation.


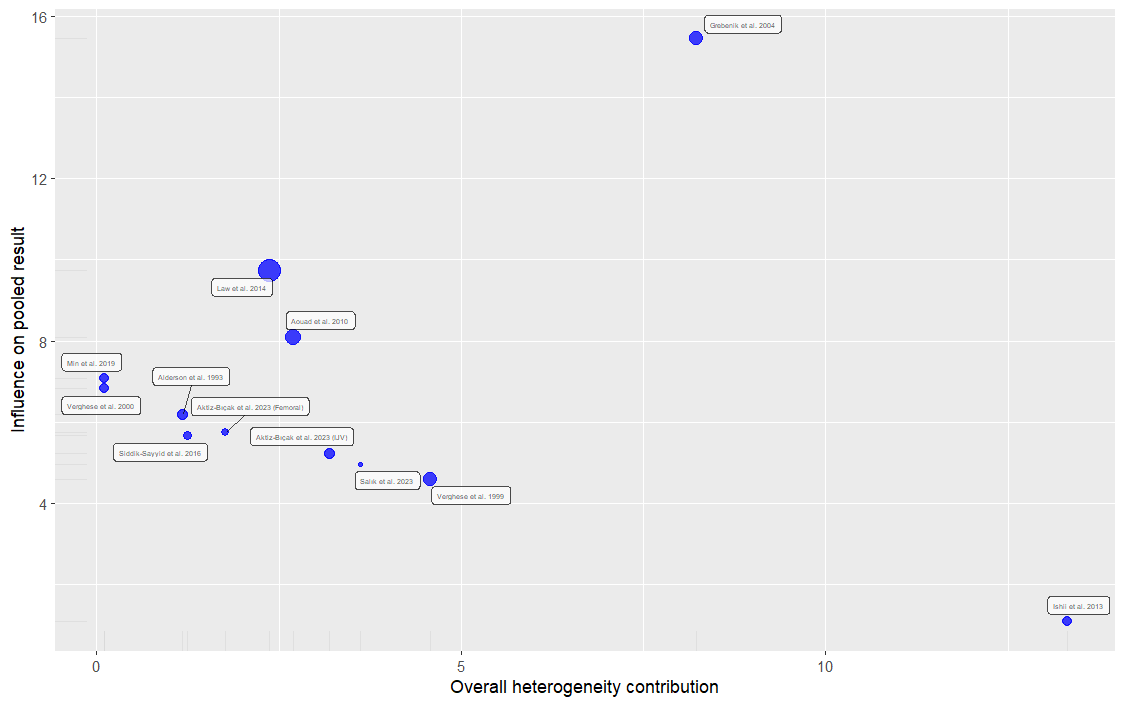


Figure S2: Baujat plot of successful cannulation (The x-axis of the Baujat plot shows the overall heterogeneity contribution of each effect size while the y-axis shows the influence of each effect size on the pooled result).


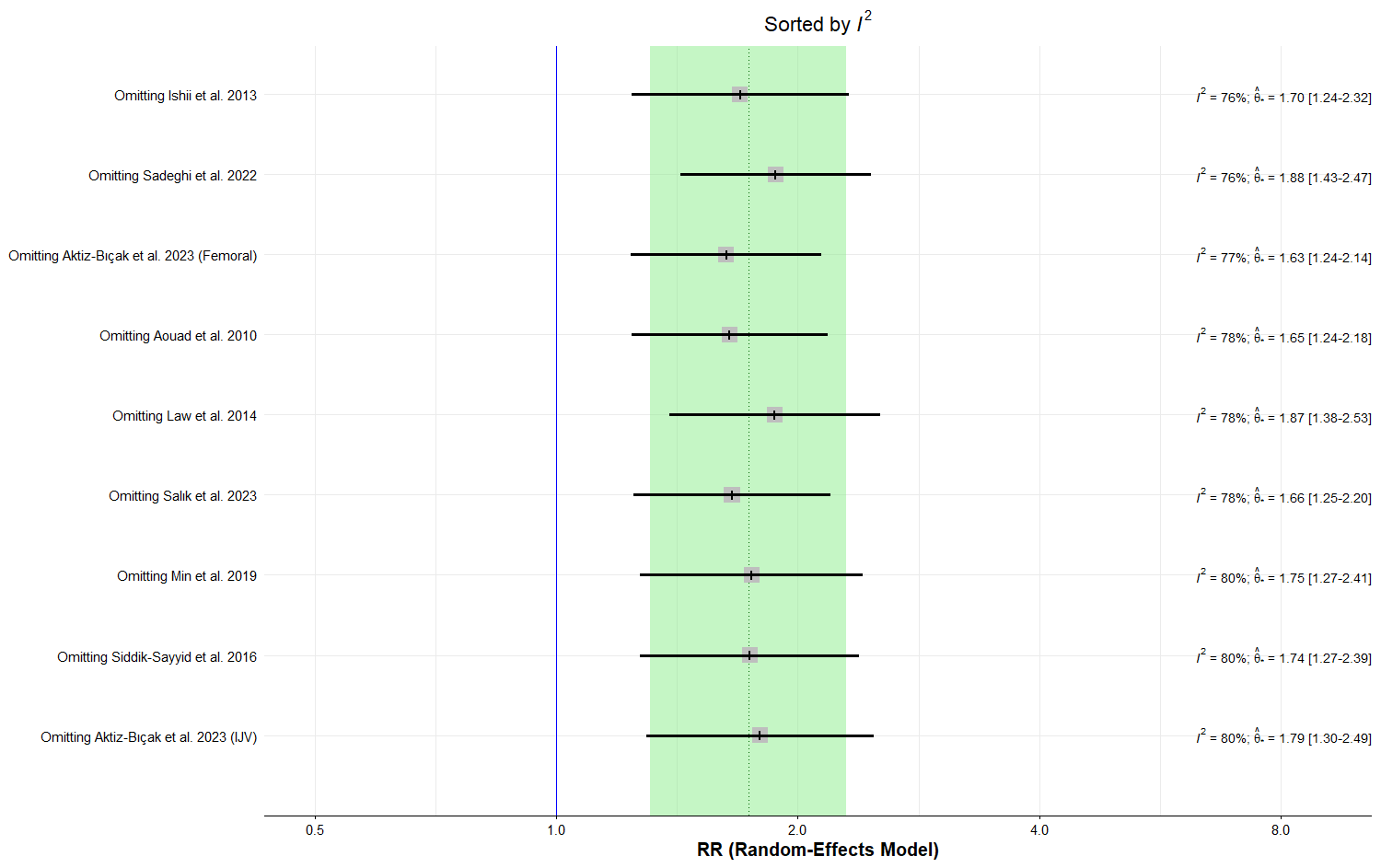


Figure S3: Sensitivity analysis of first-attempt success.


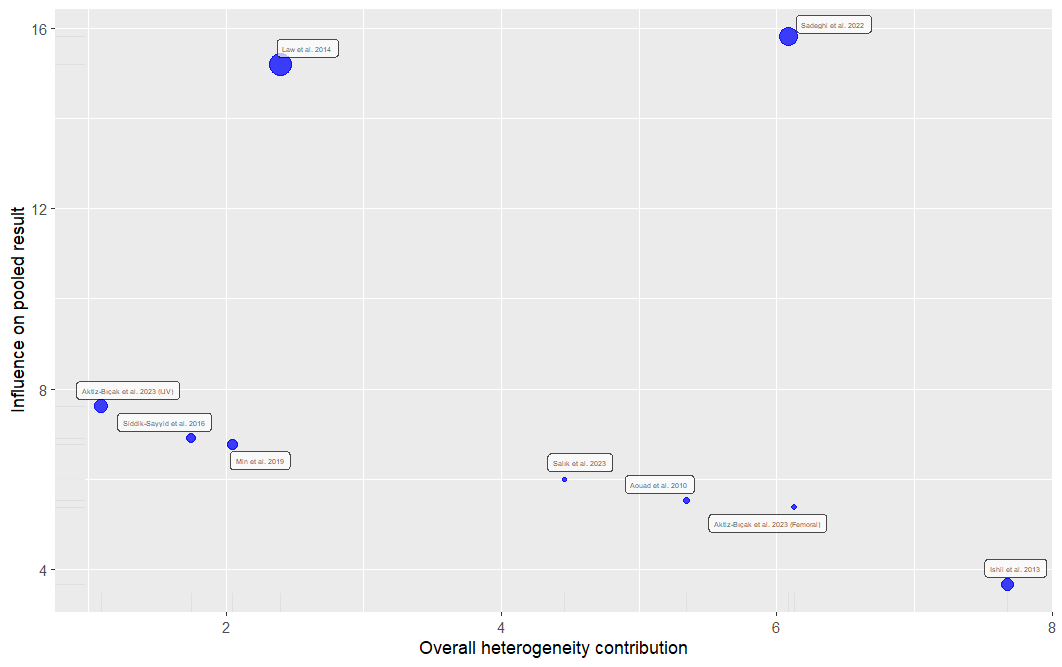


Figure S4: Baujat plot of first-attempt success (The x-axis of the Baujat plot shows the overall heterogeneity contribution of each effect size while the y-axis shows the influence of each effect size on the pooled result).


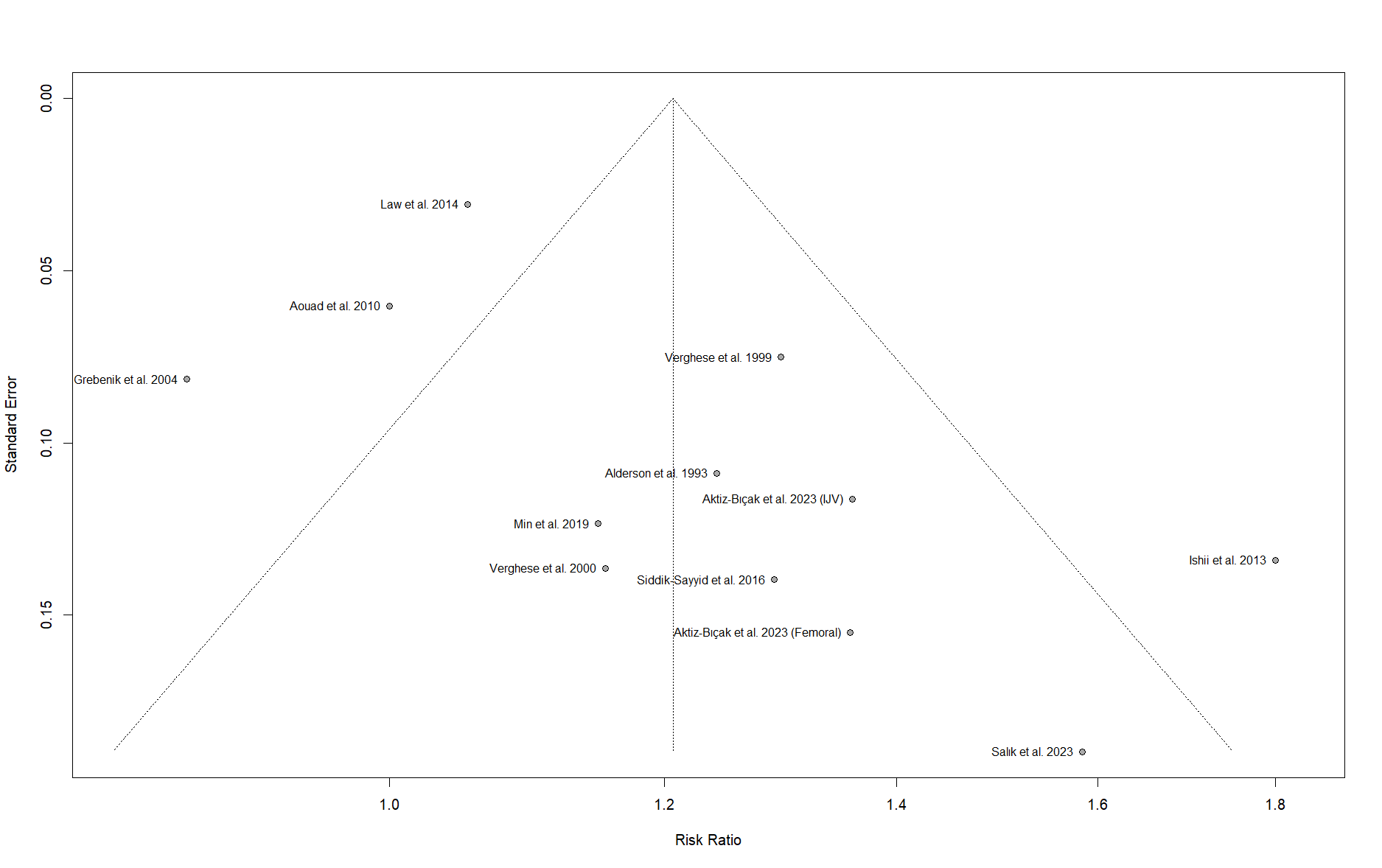


Figure S5: Funnel plot of successful cannulation.


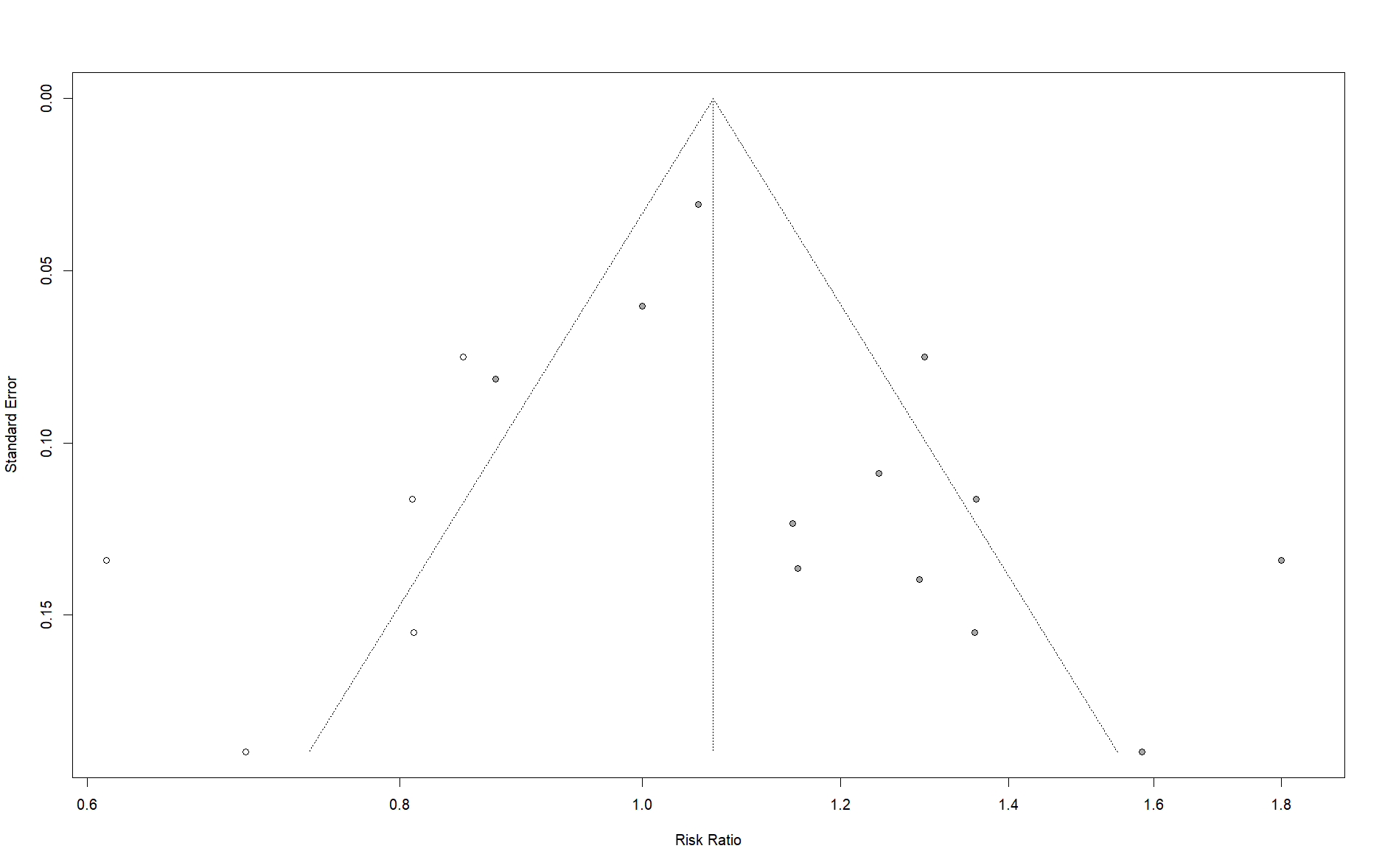


Figure S6: Trim and fill of successful cannulation.


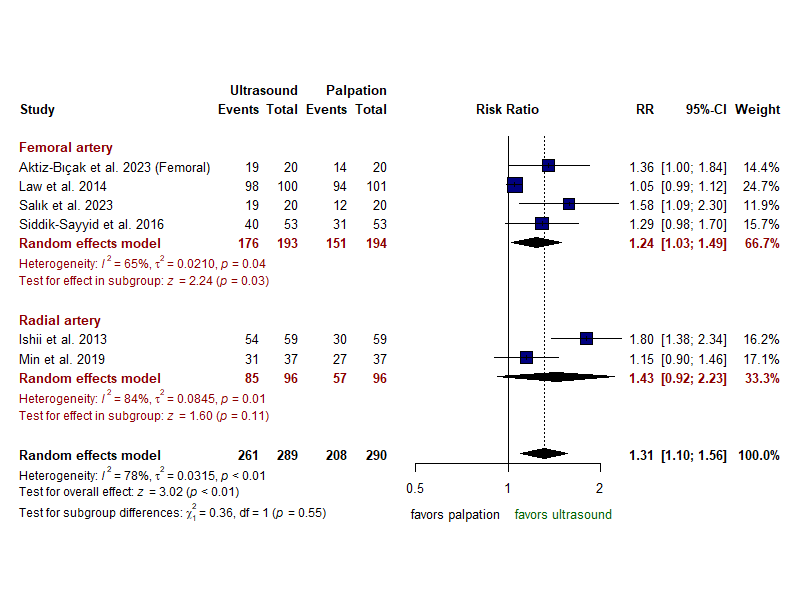


Figure S7: Subgroup analysis of successful cannulation based on the artery cannulated (Femoral Vs. Radial).


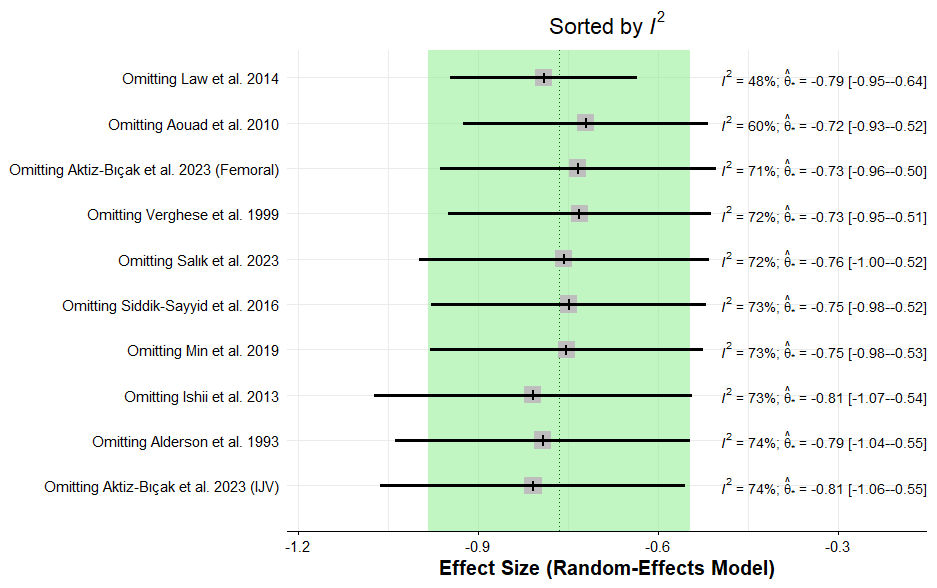


Figure S8: Sensitivity analysis of number of attempts.


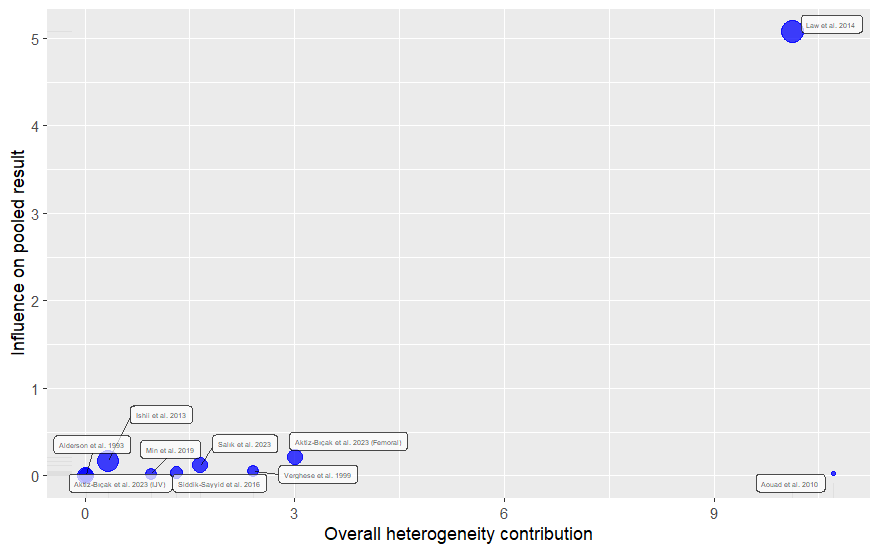


Figure S8: Sensitivity analysis of number of attempts.


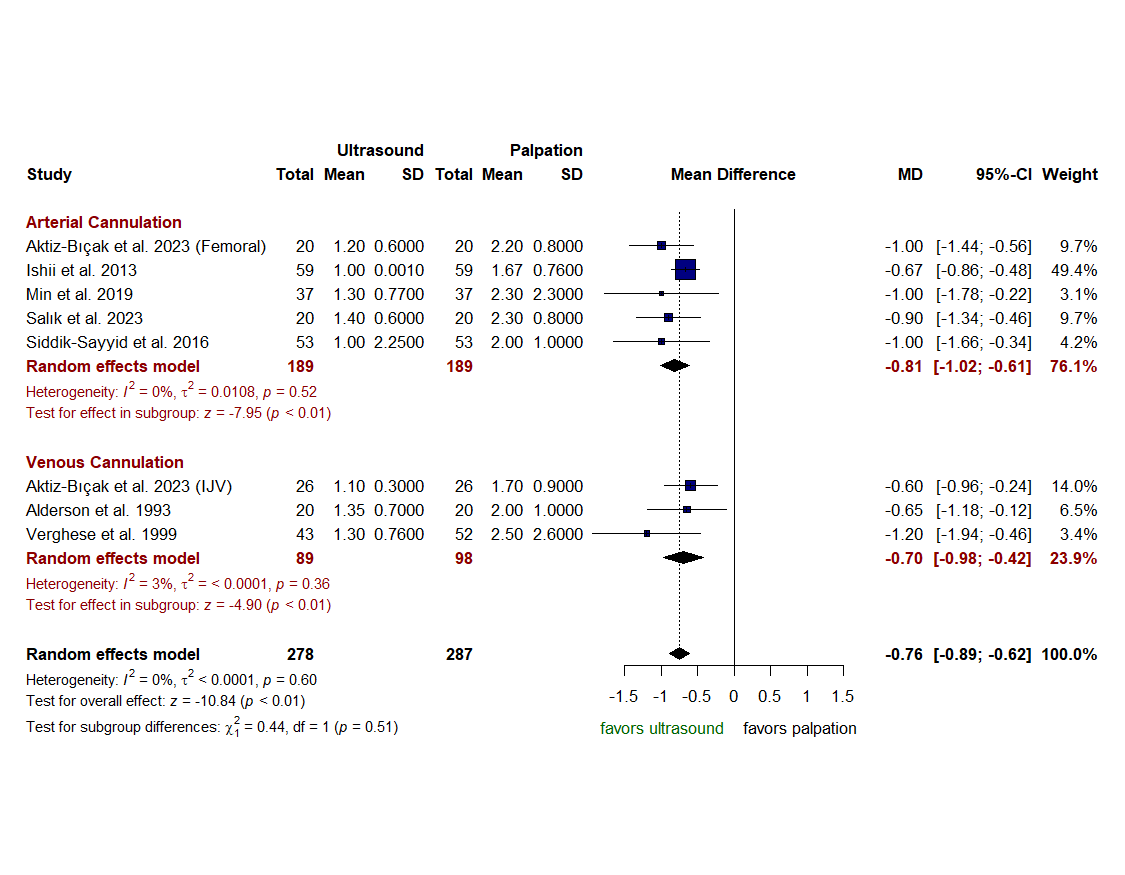


Figure S10: Results of number of attempts with outliers removed.


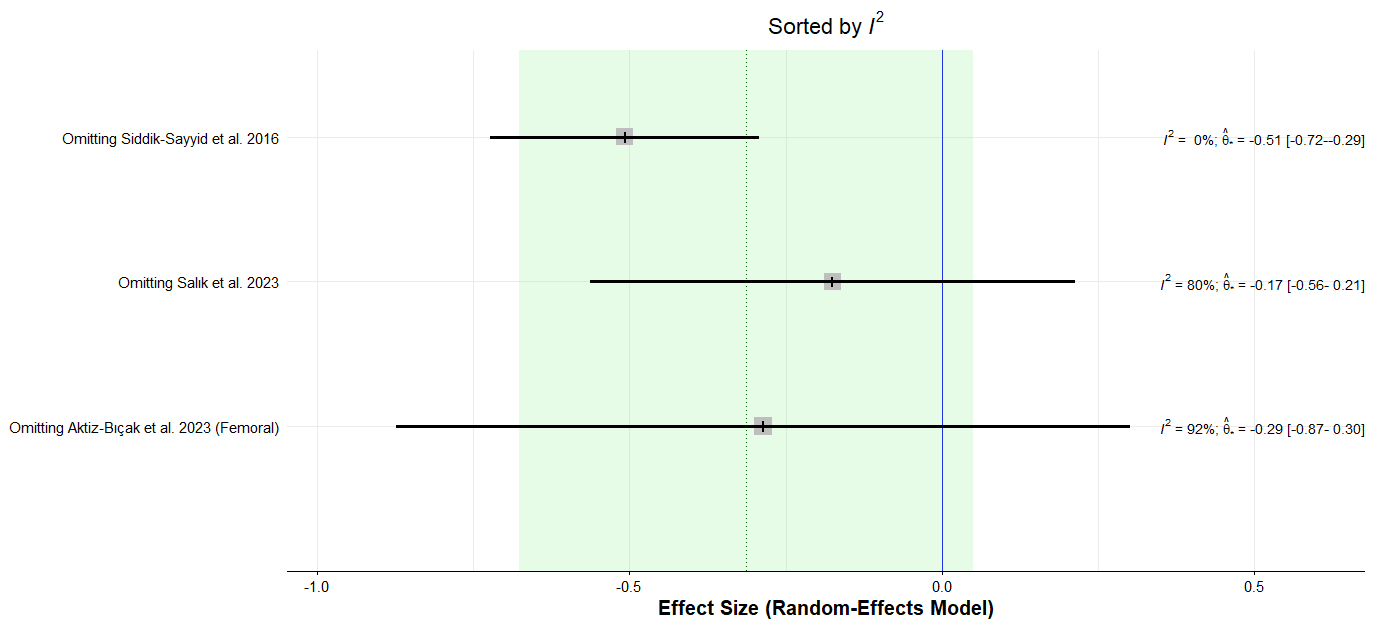


Figure S11: Sensitivity analysis of number of cannulae used.


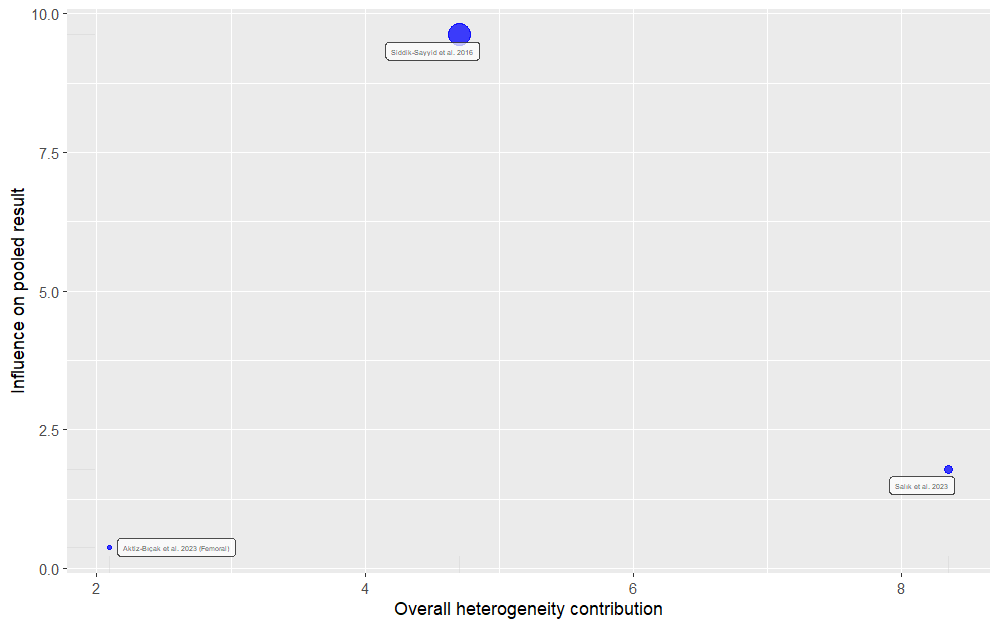


Figure S12: Baujat plot of cannulae used (The x-axis of the Baujat plot shows the overall heterogeneity contribution of each effect size while the y-axis shows the influence of each effect size on the pooled result).


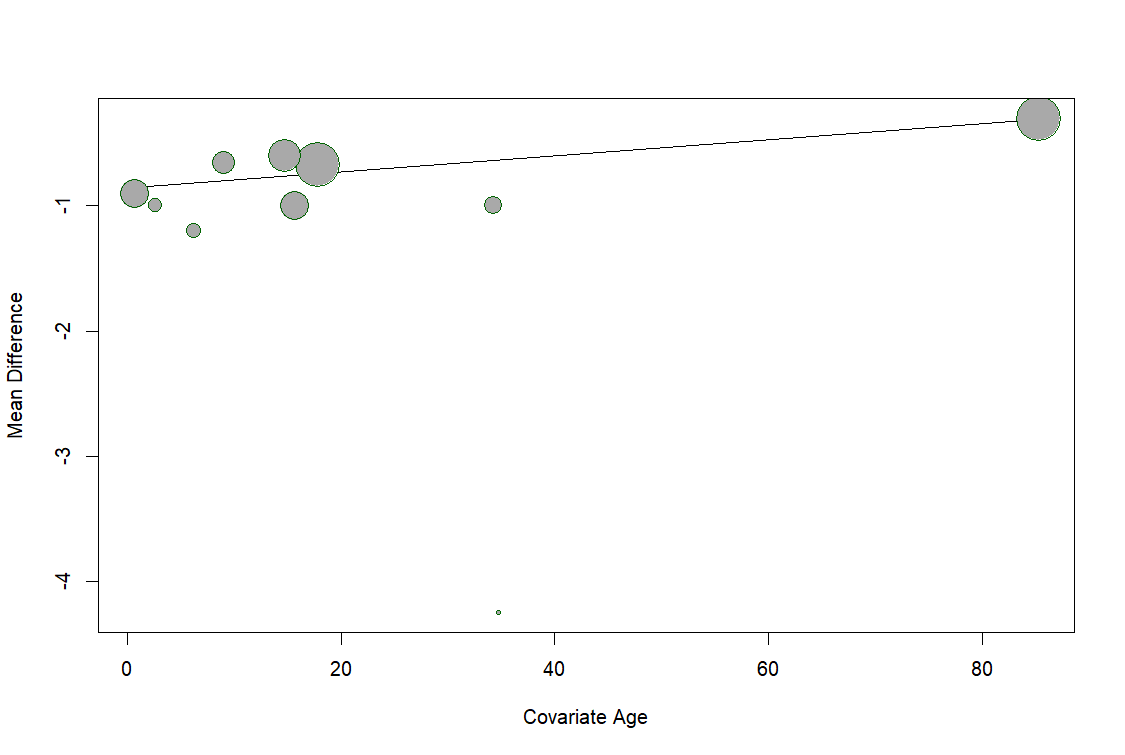


Figure S13: Bubble plot of meta-regression analysis of number of attempts based on age.


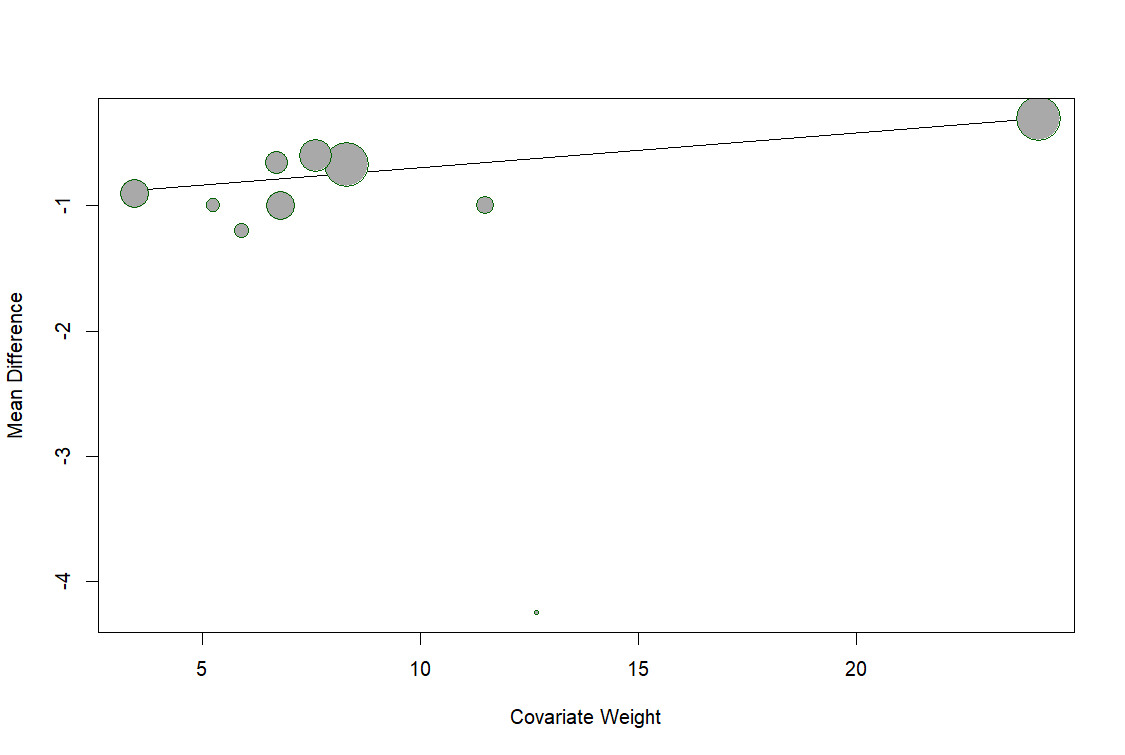


Figure S14: Bubble plot of meta-regression analysis of number of attempts based on weight.


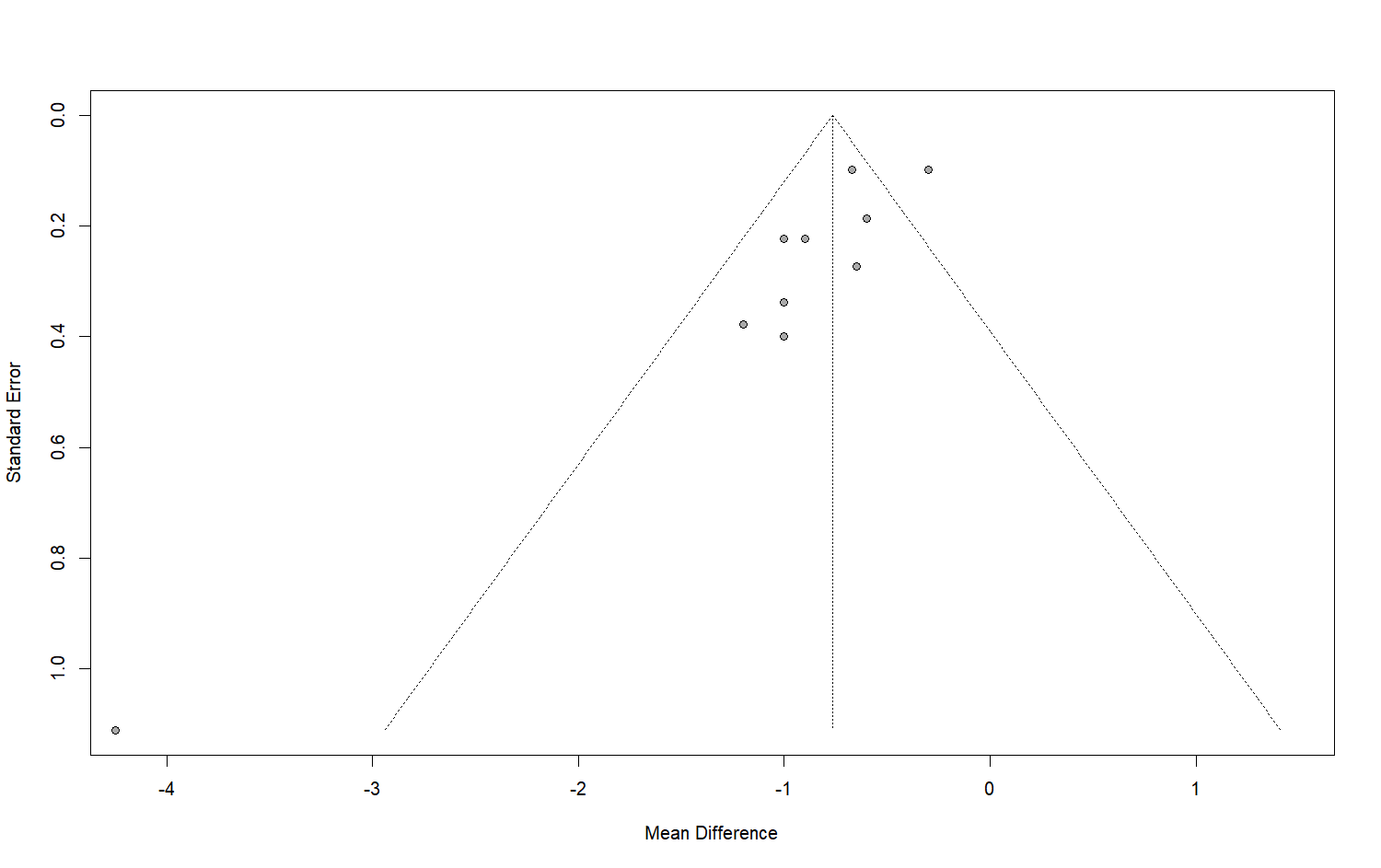


Figure S15: Funnel plot of number of attempts.


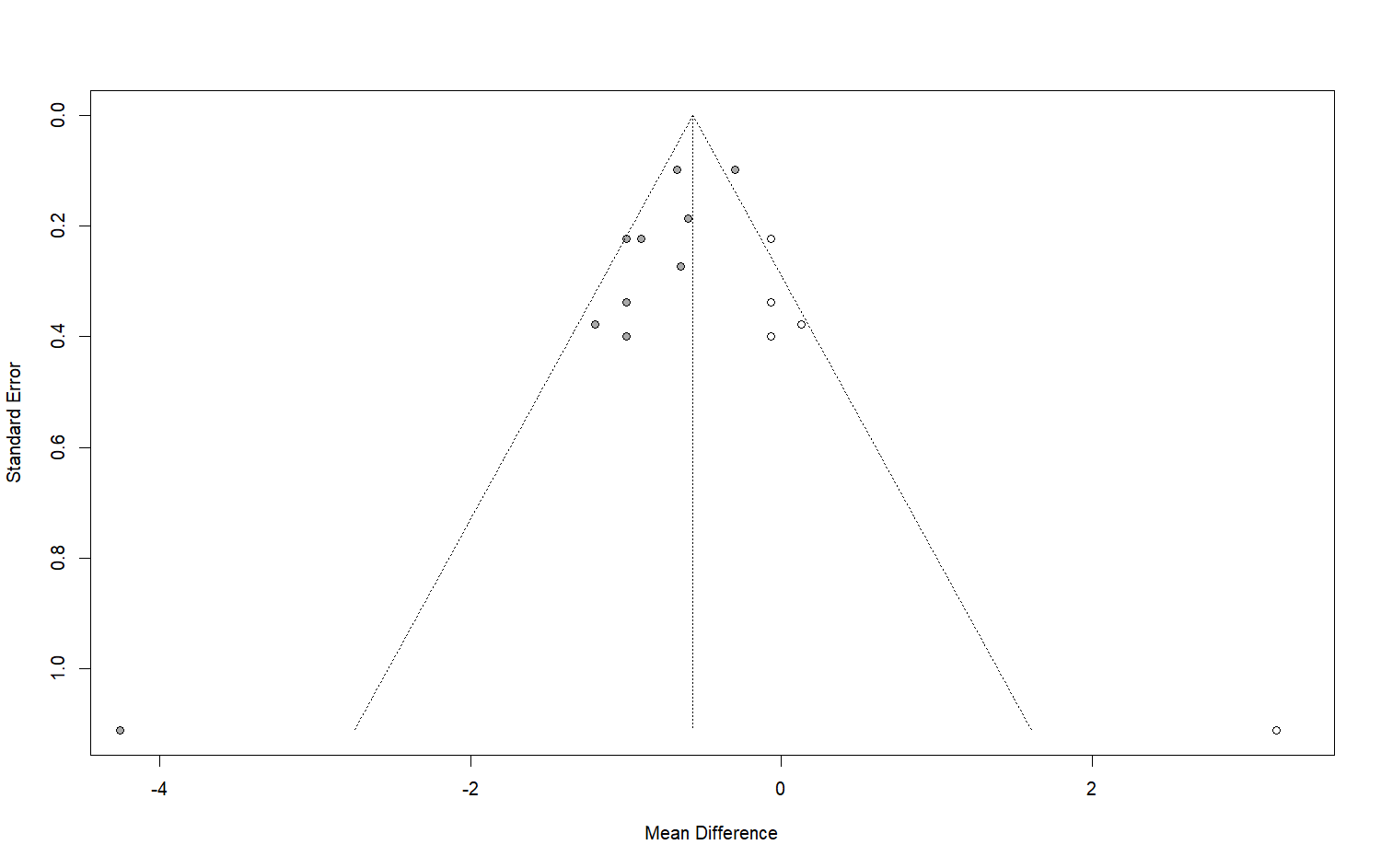


Figure S16: Trim and fill plot of number of attempts.


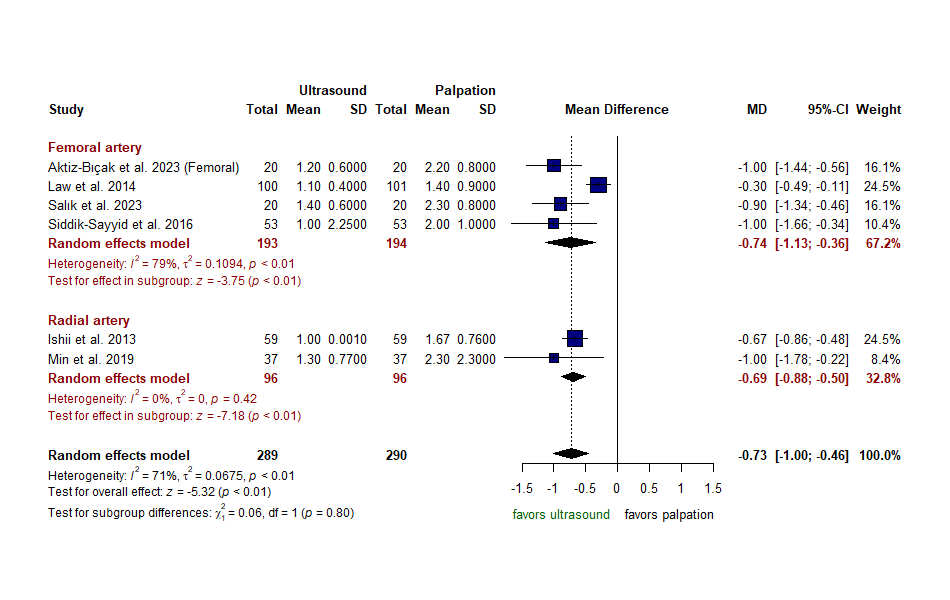


Figure S17: Subgroup analysis of number of attempts based on the artery cannulated (Femoral Vs. Radial).


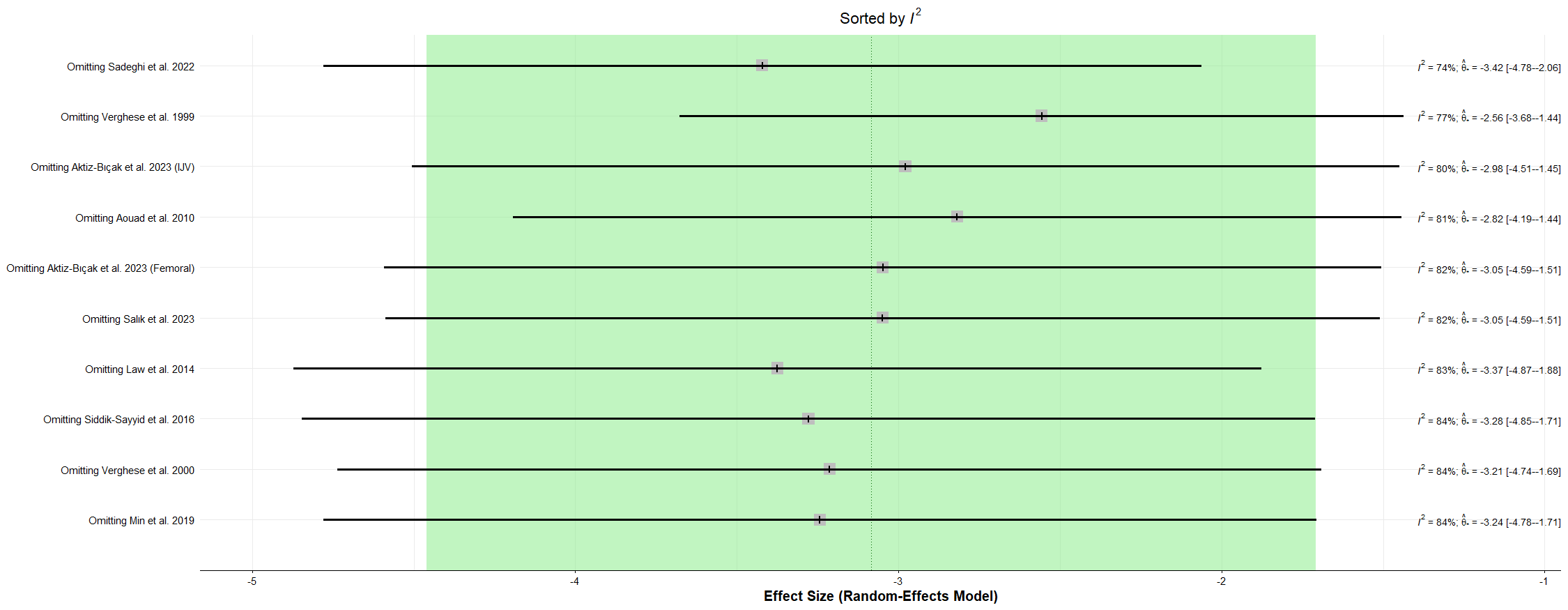


Figure S18: Sensitivity analysis of number of the time of attempted cannulation.


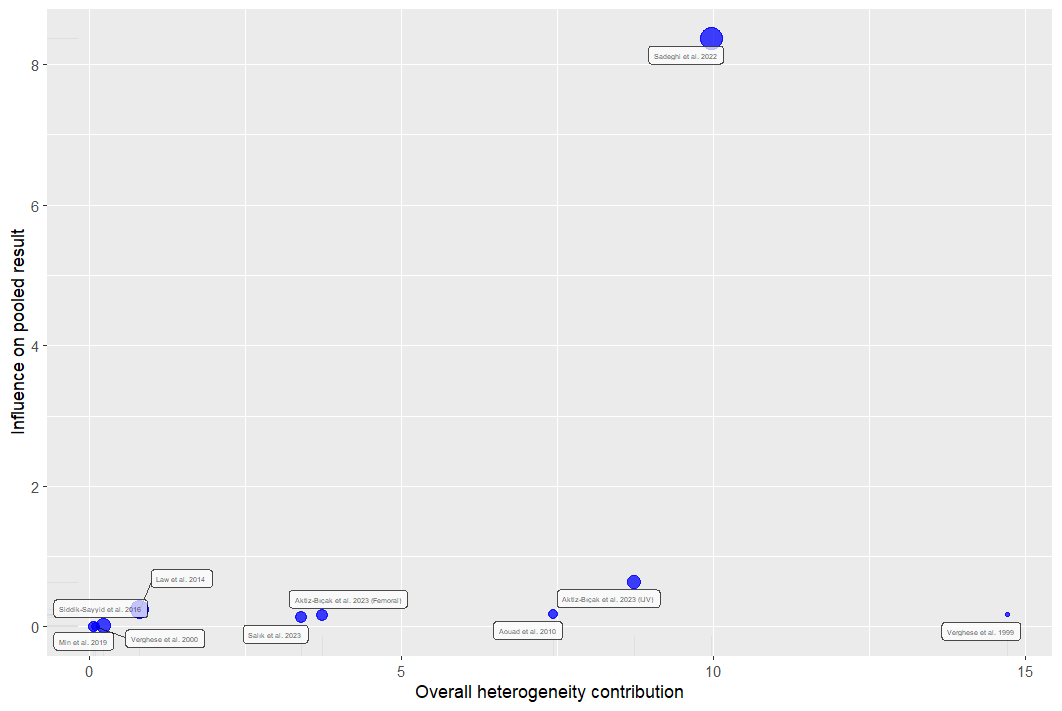


Figure S19: Baujat plot of the time of attempted cannulation (The x-axis of the Baujat plot shows the overall heterogeneity contribution of each effect size while the y-axis shows the influence of each effect size on the pooled result).


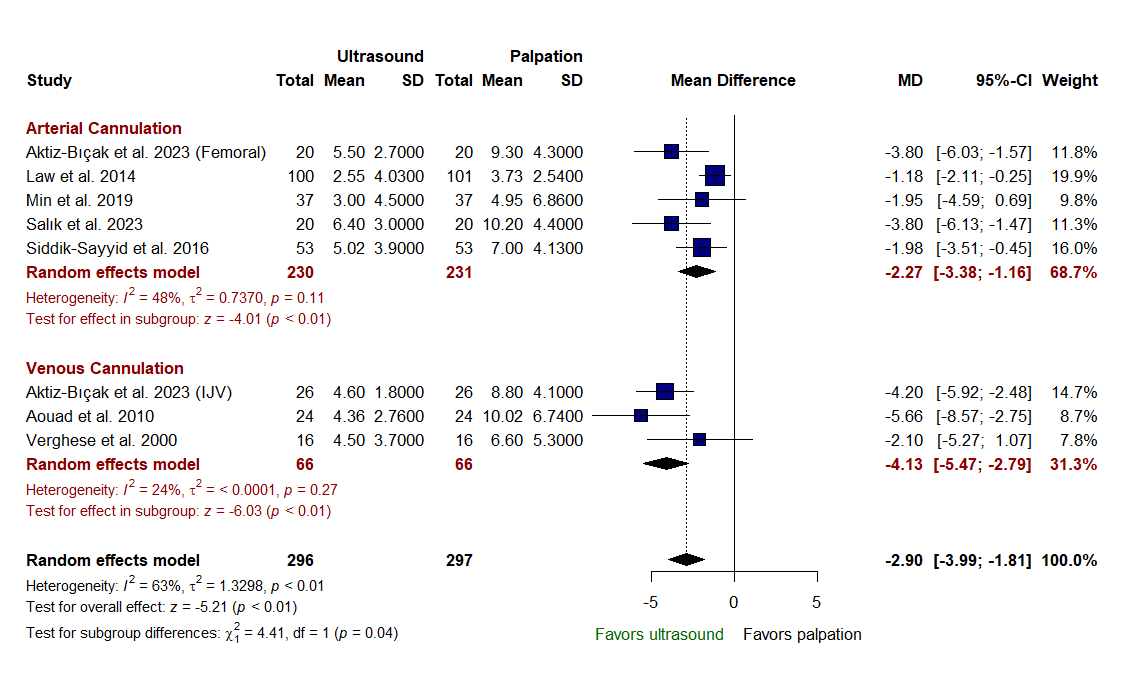


Figure S20: Results of the time of attempted cannulation with outliers removed.


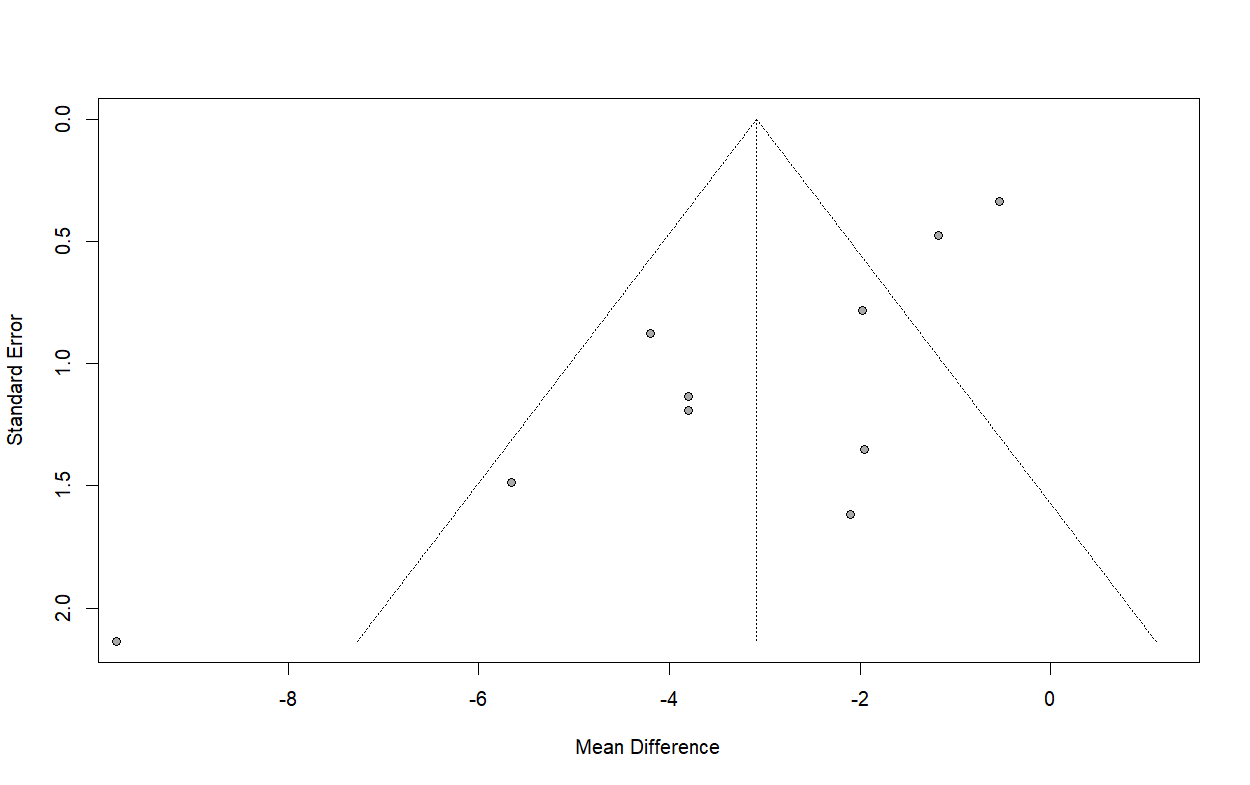


Figure S21: Funnel plot of time of attempted cannulation.


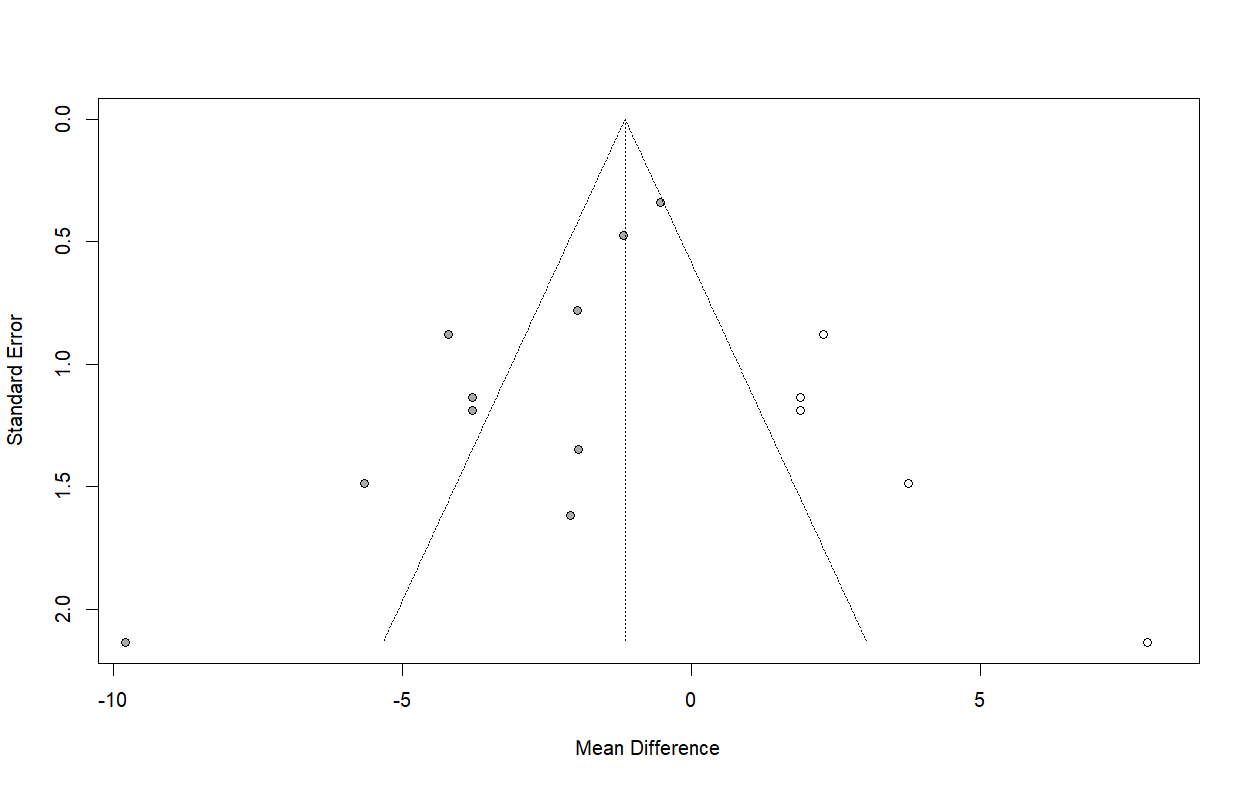


Figure S22: Trim and fill plot of time of attempted cannulation.


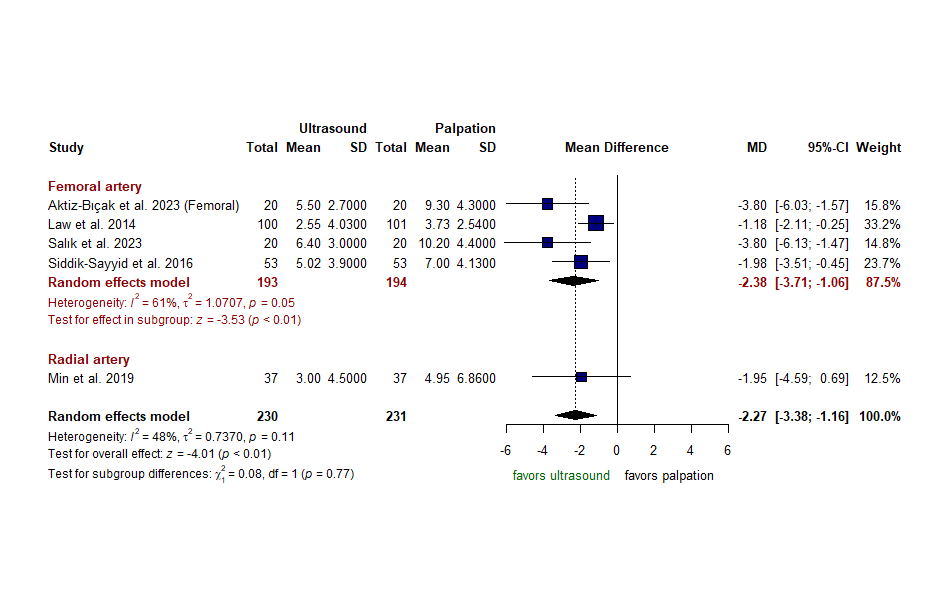


Figure S23: Subgroup analysis of time of attempted cannulation based on the artery cannulated (Femoral Vs. Radial).


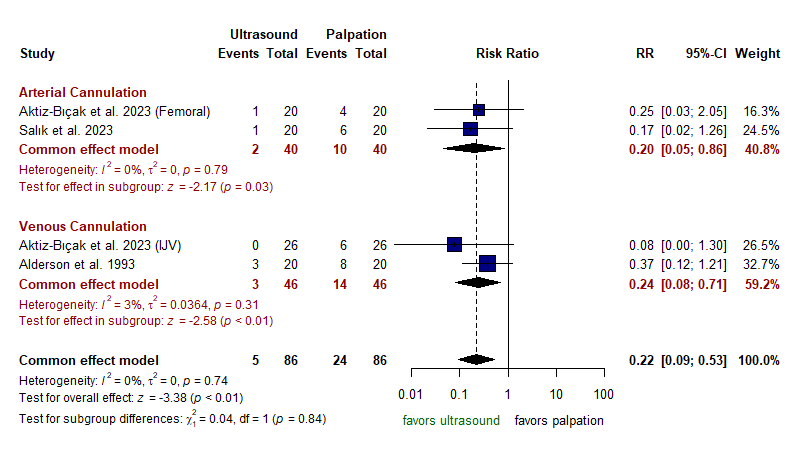


Figure S24: Forest plot of failure to pass a guide wire.


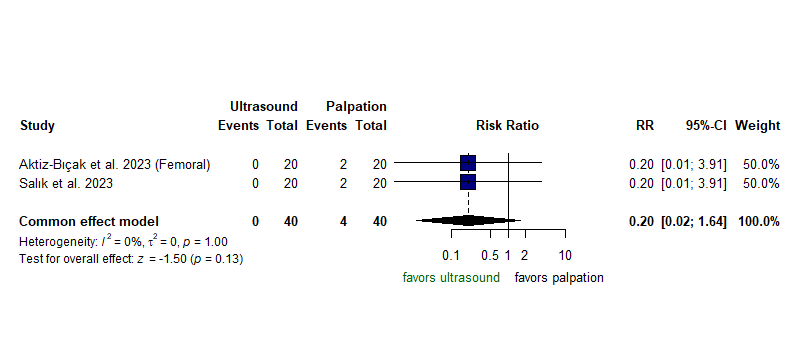


Figure S25: Forest plot of failure to puncture the vessel.


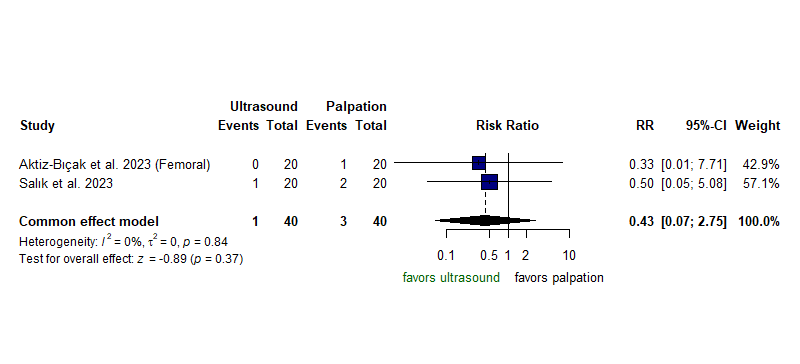


Figure S26: Forest plot of surgical cutdown.


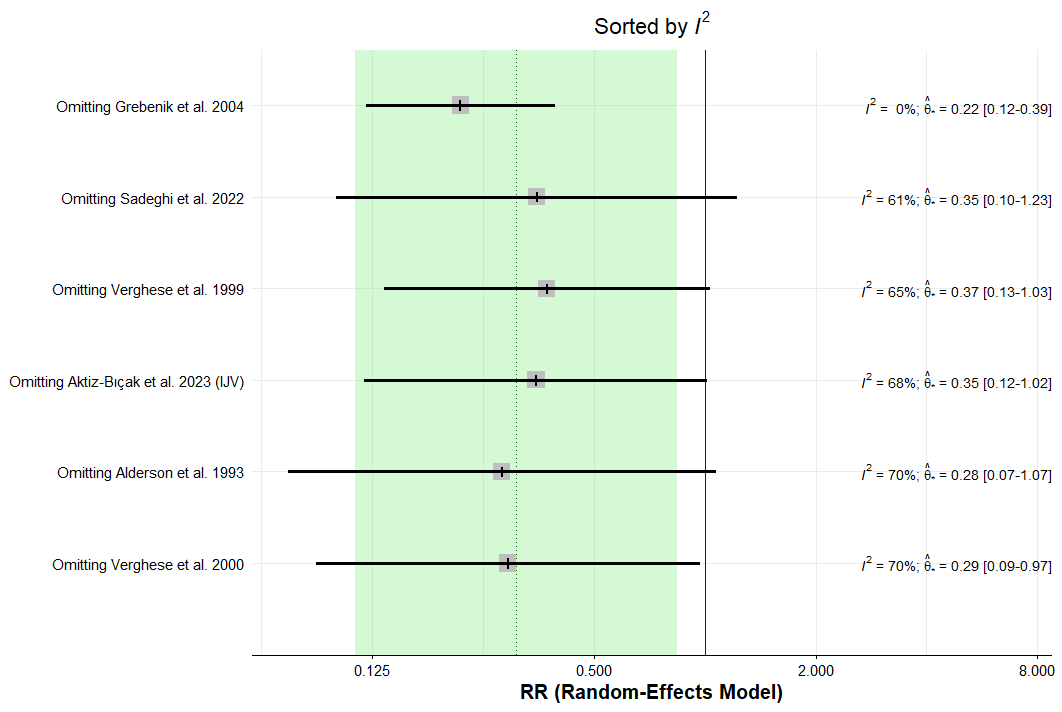


Figure S27: Sensitivity analysis of number of any complication in venous cannulation.


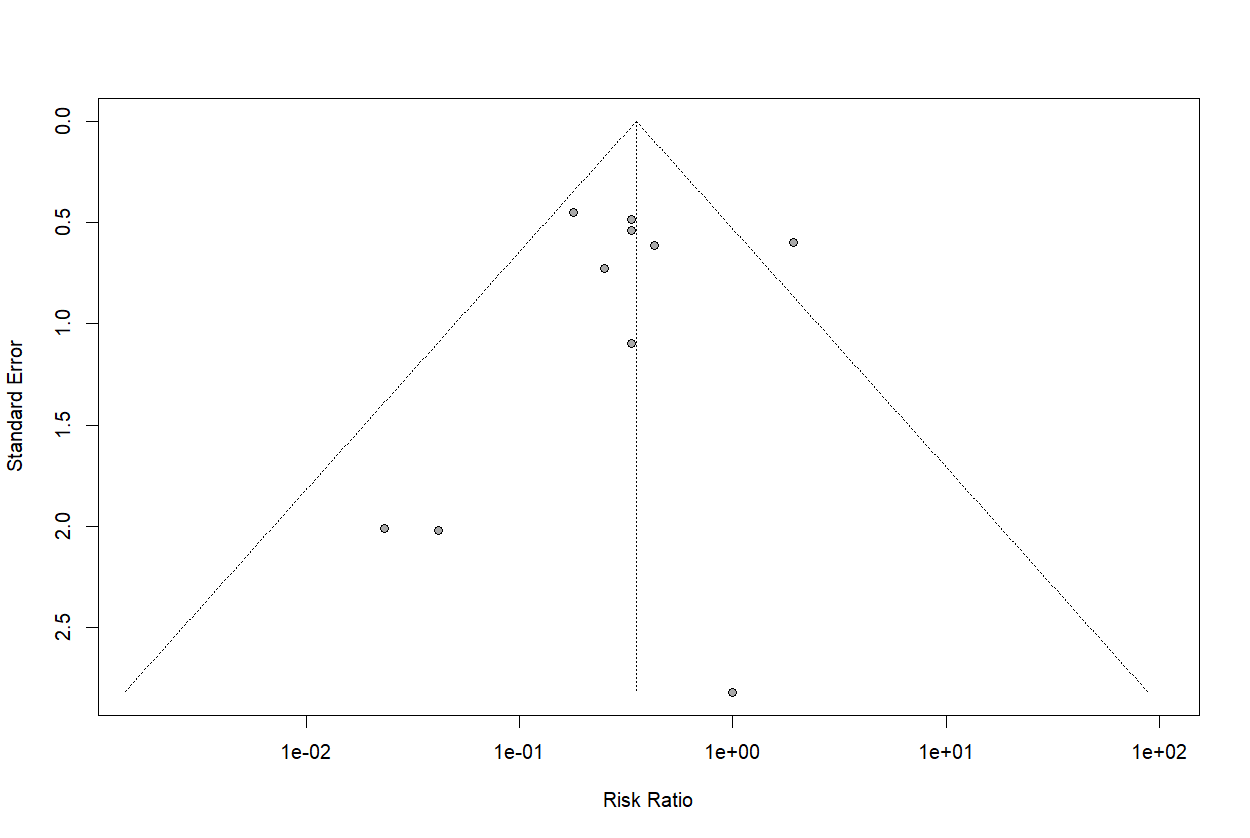


Figure S28: Funnel plot of any complication.


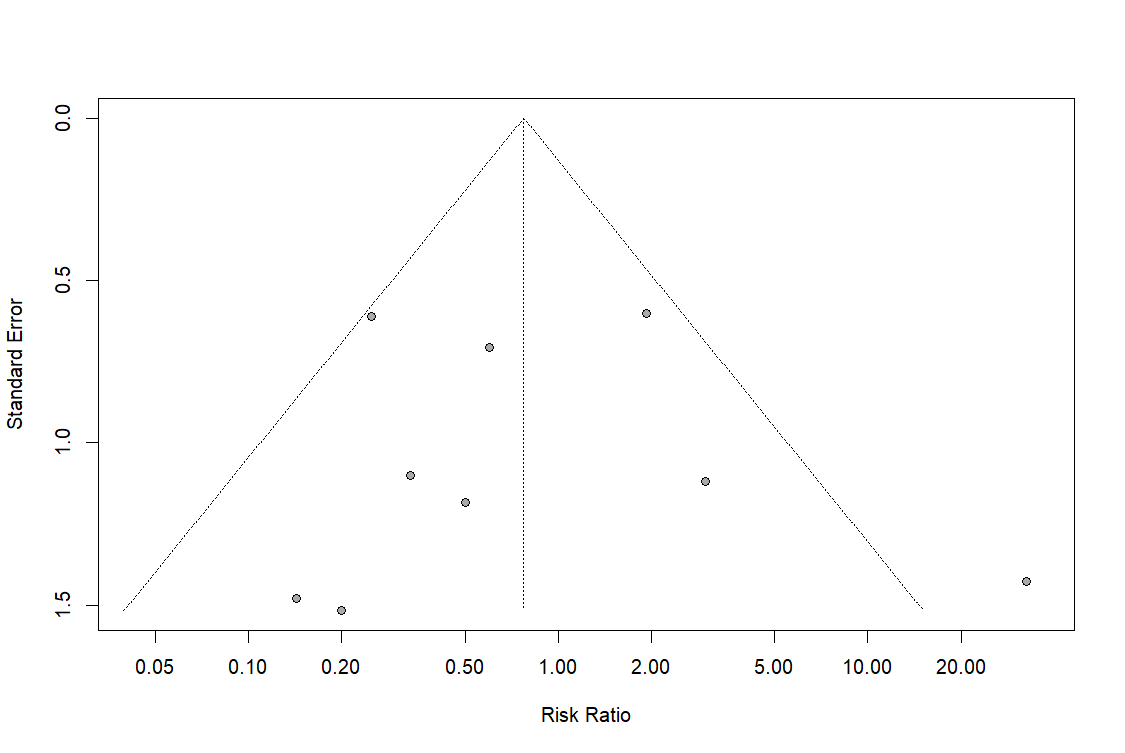


Figure S29: Funnel plot of vessel puncture.


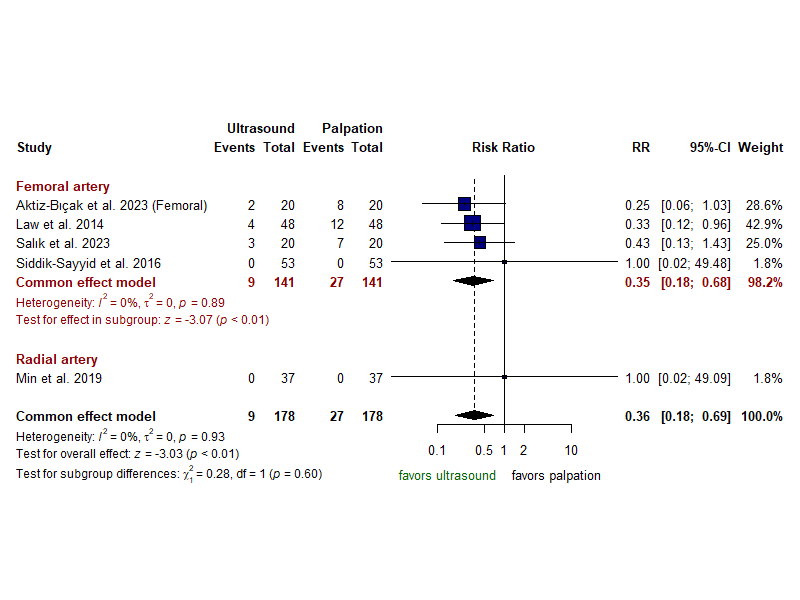


Figure S30: Subgroup analysis of any complication based on the artery cannulated (Femoral Vs. Radial).


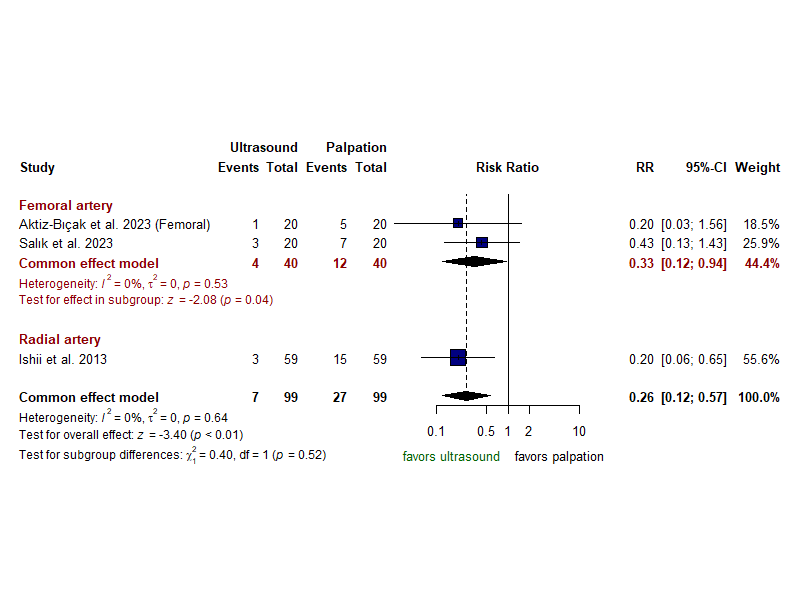


Figure S31: Subgroup analysis of hematoma formation based on the artery cannulated (Femoral Vs. Radial).
